# Nature‐Inspired Nanostructures from Multiple‐Species Biomembranes: Rational Engineering and Therapeutic Applications in Tumor‐Targeted Nanomedicine

**DOI:** 10.1002/advs.202507952

**Published:** 2025-07-02

**Authors:** Xiaodan Wei, Chi Wang, Xiangyan Chen, Yuanyuan Zhu, Mei Yang, Ningxi Li, Honglin Huang, Guangjian Zhang, Mingzhen Zhang, Yiyao Liu, Rui Gao

**Affiliations:** ^1^ Department of Thoracic Surgery the First Affiliated Hospital of Xi'an Jiaotong University Xi'an Shaanxi 710061 P. R. China; ^2^ Key Laboratory of Enhanced Recovery After Surgery of Integrated Chinese and Western Medicine Administration of Traditional Chinese Medicine of Shaanxi Province the First Affiliated Hospital of Xi'an Jiaotong University Xi'an Shaanxi 710061 P. R. China; ^3^ Department of Otorhinolaryngology Hospital of Chengdu University of Traditional Chinese Medicine Chengdu Sichuan 610072 P.R. China; ^4^ Mianyang Key Laboratory of Anesthesia and Neuroregulation Department of Anesthesiology Mianyang Central Hospital Mianyang Sichuan 621000 P. R. China; ^5^ Department of Biophysics School of Life Science and Technology University of Electronic Science and Technology of China Chengdu Sichuan 610054 P.R. China; ^6^ School of Basic Medical Sciences Xi'an Jiaotong University Xi'an Shaanxi 710061 P. R. China; ^7^ Department of Nuclear Medicine The First Affiliated Hospital of Xi'an Jiaotong University Xi'an Shaanxi 710061 P. R. China

**Keywords:** antitumor therapy, biomembrane, cell membranes, extracellular vesicles, nanostructures

## Abstract

The advent of nanomedicine is profoundly revolutionizing conventional paradigms in cancer therapeutics. Selected formulations encompassing lipid nanoparticles and polymeric nanoparticles have already attained regulatory approval for clinical treatment. Nonetheless, achieving precise spatiotemporal control of exogenous nanomedicine within intricate physiological environments remains a formidable challenge. Drawing inspiration from evolutionarily optimized natural biological architectures, the development of biomembrane‐derived nanostructures with unique biological activities provides a new impetus for designing personalized antitumor drugs. Biomembrane‐derived constituents, particularly cell membrane, extracellular vesicles, and other bioactive payloads, have inherited their precise targeting, specific tumor killing, and dynamic tumor immunosuppressive microenvironments remodeling properties in antitumor intervention. Here, the diverse biomembrane engineering strategies to equip the biomembrane with plentiful functionalities are highlighted. Moreover, the cutting‐edge innovations of mammalian cells/bacteria/plants‐derived biomembrane nanostructures and their applications for advancing cancer nanomedicine development are systematically reviewed. Finally, the current challenges and future opportunities are proposed to realize the whole potential of biomembrane nanomedicine toward clinical transformation.

## Graphical abstract

Naturally derived biomembrane nanostructures by mimicking evolutionarily optimized biological architectures could effectively suppress tumor growth and have emerged as a compelling strategy in translational biomedicine. This review provides a systematic overview of rational design and underlying antitumor therapeutic mechanisms of mammalian cells/bacteria/plants‐derived biomembrane nanostructures for advancing antitumor nanomedicine development.

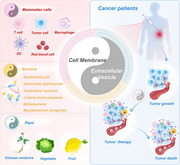

## 1 Introduction

Malignant tumor is currently a leading cause of human death globally, with an incidence of 20 million newly diagnosed cases and 10 million fatalities in 2022, which has become a severe public health challenge.^[^ ^1^ ^]^ Conventional treatments in the clinic, including surgery, chemotherapy, and radiotherapy, could eliminate tumors to a certain extent, but still have shortcomings, such as poor specificity, serious side effects, and easy relapse.^[^ ^2^ ^]^ Emerging targeted therapy and immunotherapy could improve biodistribution of antitumor drugs and generate relatively good treatment effects by targeting specific carcinogenic molecular aberrations and immune mechanisms. However, the therapeutic efficiency and patient objective response are also seriously limited by unsatisfactory target delivery efficacy, obvious off‐target toxicity, and intense drug resistance.^[^ ^3^, ^4^ ^]^ Therefore, it is essential to provide novel transformative treatment modalities for malignancies to overcome the above major challenges and improve patient outcomes.

Nanomedicine, the reasonable application of synthetic nanoscale or nanostructured materials for the medical field, especially in tumor diagnosis and treatment, has become the focus of current research due to the endowed fascinating theranostic properties.^[^ ^5^ ^]^ A multitude of synthetic nanodrugs consisting of diverse components, including micelles, liposomes, dendrimers, polymer/antibody‐drug conjugates, and inorganic nanoparticles, have been elaborated to deliver therapeutic agents better to the tumor site through passive or active targeting mechanisms.^[^ ^6^ ^]^ However, only a minority of proposed synthetic nanodrugs are currently approved in clinical patients or in clinical trials.^[^ ^7^, ^8^ ^]^ On the one hand, the “exogenous” properties of synthetic nanodrugs make them susceptible to uptake by macrophages and systemic clearance by mononeuclear phagocyte system when circulating in the blood.^[^ ^9^ ^]^ On the other hand, the synthetic nanodrugs undergo a series of rigorous human biological fluids before reaching a specific site of action, which prevents the accumulation of nanodrugs at the tumor tissue, thus limiting the effective response during drug therapy.^[^ ^10^, ^11^ ^]^ Additionally, conventional passive and active targeting methods for synthetic nanodrugs also face several barriers, such as diminished binding due to limited specific ligand modifications or off‐target effects resulting from the expression of biomarkers in normal tissues.^[^ ^12^ ^]^ There is still significant room for nanodrug improvement. A growing interest in designing novel nanodrugs based on the intrinsic characteristics of organisms is of great significance.

The imitation and innovation of natural substances and structures offers a versatile approach to designing bio‐inspired drug delivery systems. Mammalian cells/bacteria/plants, as the fundamental natural units, are pervasive and indispensable components in nature. To date, various living organisms, including mammalian cells, bacteria, plant cells, and their structural constituents, have been explored as drug delivery carriers or therapeutic agents for tumor therapy.^[^ ^13^, ^14^ ^]^ Significantly, naturally derived biomembrane nanostructures exhibited the appealing properties, including the excellent bio‐interface capabilities, great biocompatibility, and biosafety, standing out among numerous biomimetic approaches.^[^ ^15^ ^]^ Natural biomembranes are predominantly derived from two biological sources: isolated cell membranes (CMs) and extracellular vesicles (EVs). Previous studies have substantiated that membrane fragments and EVs still preserve respective membrane proteins and inherent biological functionalities even after separation from the cellular source, whereas these properties are difficult to completely replicate by artificial synthetic materials.^[^ ^16^ ^]^ It is reported that genetically engineered cells could enrich specific small interfering RNAs (siRNA) in EVs, resulting in over a tenfold enhancement in functional siRNA delivery efficiency to mice compared to synthetic lipid‐based nanocarriers.^[^ ^17^ ^]^ Collectively, biomembrane nanostructures are highly efficient delivery tools, providing intrinsic targeting, multiplex cargo loading, immune evasion, biological barrier penetration, among other advantages. Recently, integrating native biomembranes with synthetic materials to construct biomimetic nanoplatforms has attracted growing attention. This biohybrid approach significantly improves the in vivo pharmacokinetics of nanodrugs by prolonging circulation half‐life and diminishing immunogenicity.^[^ ^15^ ^]^ Moreover, the development of biomembrane engineering technologies has further strengthened their original functions or endowed them with additional functionalities, thus allowing biomembrane‐based nanodrugs to show more various performances in tumor treatment.^[^ ^18^ ^]^

**Figure 1:**
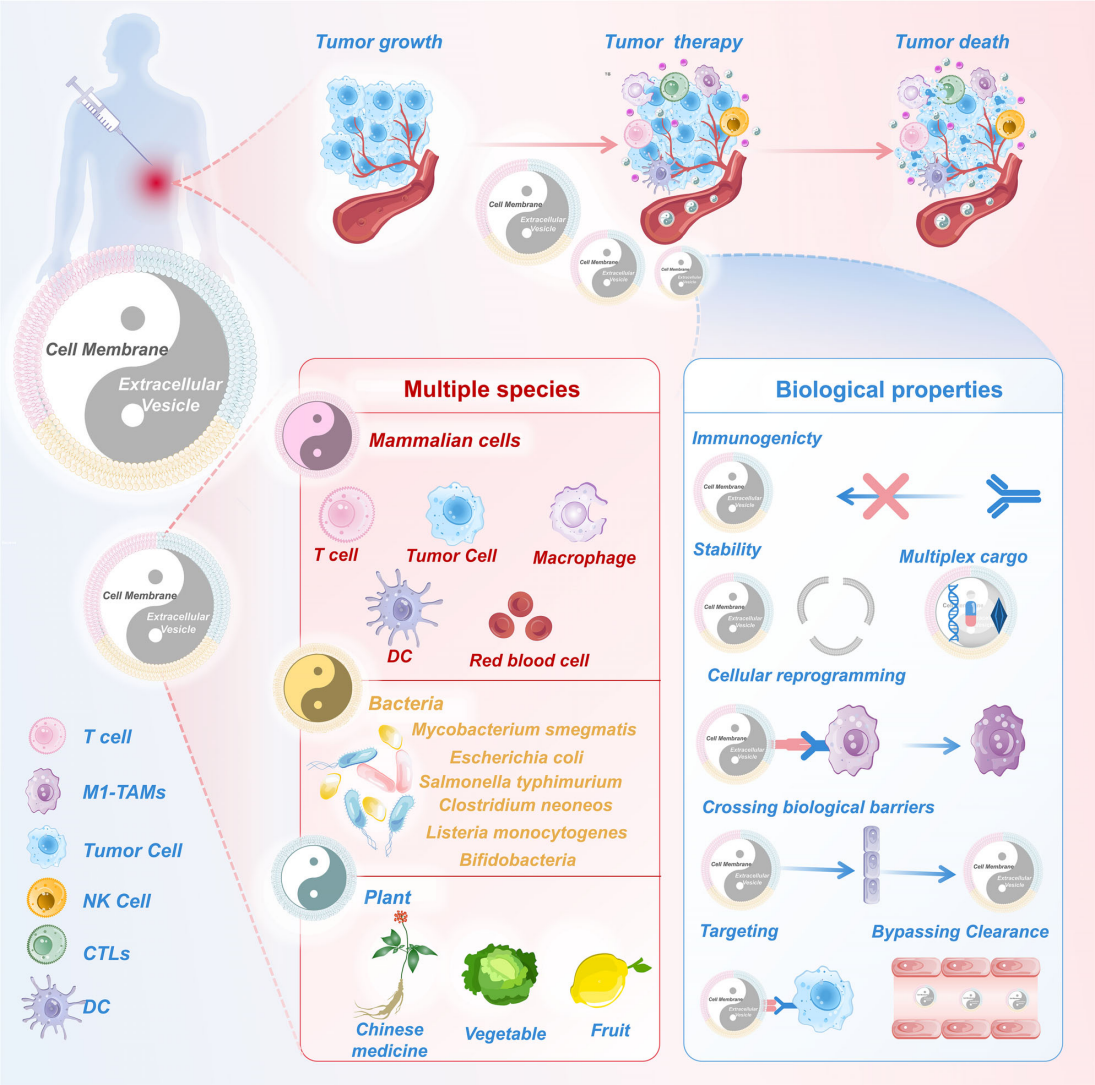
Schematic illustration of bioinspired nanomedicine based on mammalian cells, bacteria, and plants‐derived biomembrane nanostructures for precise antitumor therapy.

Herein, we systematically summarize the latest advances in treatment strategies, including biomembrane nanostructures derived from mammalian cells, bacteria, and plants, aimed at the rational design of personalized nanomedicine platforms for cancer therapy (**Figure** **1**). The preparation methods and engineering technologies of biomembrane nanostructures are first introduced. Subsequently, we detail the diverse kinds of biomembrane nanostructures using cell classification as the central line and spotlight their promising applications in drug delivery. Finally, the prospects and challenges of biomembrane nanostructures in antitumor applications are addressed for further clinical translation. We expect that the review can provide a valuable perspective for researchers to design novel biomembrane nanomedicines for cancer treatment.

## 2 Strategies Guidance for Preparing and Engineering Biomembrane Nanostructures

The transport fate of biomembrane nanostructures in vivo is governed by their inherent biological properties. A thorough understanding of their biology, drug‐loading mechanisms, and cellular delivery is crucial for developing these biomimetic nanostructures as next‐generation delivery platforms. This section details the preparation of CMs‐ and EVs‐based nanostructures, including isolation, purification, and drug‐loading approaches (summarized in **Table** **1**), while also summarizing mainstream engineering strategies for cancer therapy.

**Table 1:** Extraction methods and antitumor therapeutic applications of biomembrane nanostructures.

Cell types	Source	Membrane extraction method	Nanomedicine	Cancer type	Refs.
Mammalian	RBC	Hypotonic lysis	RBC(IR)‐NDs	Breast cancer	[144]
	Platelet	Freeze‐Thaw Cycles	MSN@PM‐C‐A	Liver cancer	[145]
	Macrophage	Hypotonic lysis, differential centrifugation	PMMNPs	Colorectal cancer	[146]
		Hypotonic lysis, ultrasonication, differential centrifugation	IL12/CSF1R‐MM‐IRC18‐LPS	Glioblastoma	[147]
	Neutrophil	Ultrasonication, differential centrifugation	Neu/MSC‐sEVs	Gastric cancer	[148]
	NK	Differential centrifugation, polymer‐based precipitation	mNK‐sEV	Gastric cancer	[149]
	Stem cell	Differential centrifugation	HMnO_2_‐MSC‐TAT@PTX	Lung cancer	[79]
	Cancer cell	Hypotonic lysis, differential centrifugation	MPPC@CM	Breast cancer	[150]
Bacteria	*Mycobacterium smegmatis*	Enzymatic lysis, sonication, organic solvent extraction	PC7A/CpG@BM‐Mal	Neuroblastoma, melanoma	[151]
	*Escherichia coli*	Enzymatic lysis, hypotonic lysis, differential centrifugation	HM‐NPs	Breast cancer, colorectal cancer	[152]
	*Escherichia coli*	Ultrafiltration concentration, ultracentrifugation	DOX/Ce6‐OMVs@M	Breast cancer	[153]
	*Escherichia coli*	Ultrafiltration concentration, ultracentrifugation	Angiopep2‐OMVs@PTP/47aD	Glioblastoma	[154]
Plant	*Platycodon grandiflorum*	Differential centrifugation, density gradient centrifugation	PGEVs	Breast cancer	[129]
	*Curcuma longa*	Differential centrifugation, ultracentrifugation	DR5‐CNV/DOX	Breast cancer	[155]
	*Lemon*	Differential centrifugation, density gradient centrifugation	HRE‐DOX	Ovarian cancer	[134]
	*Spinach*	Tissue homogenization, differential centrifugation	TK/PLT@Lipo‐HMME/Zeb	Colorectal cancer	[28]
	*Hypericum perforatum*	Size exclusion chromatography, ultracentrifugation	HPDENs	Melanoma	[124]

### 2.1 CMs‐Derived Nanostructures

Natural CMs from mammalian, bacterial to plant cells are worthy of being studied owing to their significantly optimized characteristics during the evolutionary process.^[^ ^19^, ^20^ ^]^ The intricacy and dynamism of CMs exceed just being a passive phospholipid bilayer sheath. Membrane‐spanning proteins and carbohydrates are active constituents of cellular mechanisms, acting as the first responders to the microenvironment that surrounds the cell. The majority of the targeting and biointerface interaction capabilities (such as adhesion interactions, binding, and receptor‐ligand) of a cell could be ascribed to the cytoplasmic membrane.^[^ ^21^ ^]^ Currently, CMs‐derived nanostructures have emerged as an interesting biomimetic nanodrug based on cell function‐driven strategies for in vivo applications.^[^ ^22^, ^23^ ^]^

The fabrication of CMs‐based nanodrugs typically implicates cell rupture, membrane extraction, and membrane camouflaging. Hinging on the innate nature of diverse cells, the membrane extraction procedures are dissimilar. For mammalian anucleate cells, the operation of membrane extraction is relatively straightforward; hypotonic lysis and freeze‐thaw cycles are generally used to thoroughly destroy the outer membranes and remove the intracellular contents.^[^ ^24^, ^25^ ^]^ However, much more complicated extraction protocols are required during the mammalian nucleated CMs extraction process. Hypotonic lysis, mechanical grinding, freeze‐thaw cycle‐erosion, and discontinuous sucrose gradient centrifugation could be performed to separate pure empty membranes from other cell remnants (cytoplasm and cell nuclei).^[^ ^26^ ^]^ Due to distinct cell wall structures, bacterial membrane (BM) extraction strategies diverge significantly between gram‐negative and gram‐positive species. For gram‐negative bacteria with an outer membrane and a thin peptidoglycan layer, gentle lysozyme treatment degrades peptidoglycan, followed by ultracentrifugation to collect outer membranes; inner membranes are isolated through density gradient centrifugation, with polymyxin B affinity chromatography often used to remove endotoxin. Gram‐positive bacteria, lacking an outer membrane but featuring a thick peptidoglycan layer, require treatment with lysozyme combined with sonication for cell disruption, followed by differential centrifugation and density gradient purification.^[^ ^27^ ^]^ Plant cell membrane extraction requires enzymatic degradation of the rigid cell wall using cellulase and pectinase to generate protoplasts, followed by osmotic lysis in hypotonic buffer. Membrane fractions are isolated via differential centrifugation and purified through sucrose density gradient centrifugation.^[^ ^28^ ^]^ Obtained membrane fragments are washed by precooled isoionic buffer and stored at −80 °C in an endotoxin‐free solution containing protease inhibitors to maintain the long‐term stability of membrane protein activity. Loading therapeutic agents onto the membrane shell or into the nanocore is the most essential and ultimate step that determines the optimal preparation of biomembrane nanostructures. A series of methods have been applied to obtain CMs‐derived nanostructures, including coextrusion, sonication, freeze‐thaw cycling, microfluidic electroporation, and membrane binding approaches.^[^ ^29^, ^30^, ^31^ ^]^

### 2.2 EVs‐Derived Nanostructures

EVs are a kind of nano‐biological particle with lipid membrane structure generated by cells, which were initially considered more like carriers of metabolites or cell debris. Over the past decade, more and more evidence has suggested that EVs mediate cell‐cell communication, participate in the regulation of various physiological and pathological processes, and possess a multitude of biological functions.^[^ ^32^, ^33^ ^]^ Almost all natural cells, including mammalian, bacterial, and plant cells, could produce EVs.^[^ ^34^ ^]^ For mammalian cells, EVs could be roughly divided into exosomes and microvesicles according to the biogenetic pathways.^[^ ^35^ ^]^ Exosomes, ranging from 30 to 200 nm in diameter, are generated through the fusion of multivesicular bodies with the plasma membrane. Micro‐vesicles are produced by directly budding outward from the plasma membrane, with a diameter of 100–1000 nm.^[^ ^36^ ^]^ Apart from exosomes and micro‐vesicles, mammalian cells also generate a few vesicle bodies related to diverse types of cell death, including apoptotic bodies produced from cells during apoptosis or the dead process with the size of 1–5 µm, pyroptotic inflammatory vesicles released during pyroptosis and necrotic vesicles released by necrotic cells.^[^ ^37^ ^]^ Gram‐negative bacteria produce outer membrane vesicles (OMVs) through outward budding followed by traversing the thick peptidoglycan layer. In contrast, gram‐positive bacteria generate membrane vesicles via direct budding from the cytoplasmic membrane. Plants secrete EVs through three main pathways: the multivesicular body (MVB) pathway, exocyst‐positive organelle‐mediated pathway, and vacuole‐derived trafficking, with the MVB pathway being the predominant route.^[^ ^38^ ^]^ Collectively, the surface markers and encapsulated components (such as lipids, proteins, miRNAs and so on) of EVs from different sources have obvious differences, EVs inherit biological properties from their deriving source and reflect the biocomponent of donor cell, enabling it to spontaneously target certain cells and achieve excellent biocompatibility with living organisms, which have great potential in the field of drug delivery.^[^ ^39^, ^40^ ^]^

Classical differential ultracentrifugation is considered the benchmark for EVs separation, which could separate EVs from various cells or biofluids by employing diverse centrifugal forces and durations. Recently, there have also been several emerging technologies for separating EVs, such as specific isolation kits, polymer‐based precipitation, ultrafiltration, size exclusion chromatography, immunoaffinity chromatography, and so on.^[^ ^41^ ^]^ Actually, multiple methods are combined to improve the efficiency and purity of EVs separation. For example, the differential ultracentrifugation method is first used to remove most of the impurities, and then the size exclusion chromatography method is employed to further purify the EVs. Similar to CMs, EV‐derived membranes could be extracted and modified on synthetic nanomaterial surfaces. Apart from being able to serve as a coating biomaterial for drug delivery, EVs can also act as direct therapeutic agents for antitumor therapy.

### 2.3 Strategies for Engineering Biomembrane Nanostructures for Cancer Therapy

**Figure 2:**
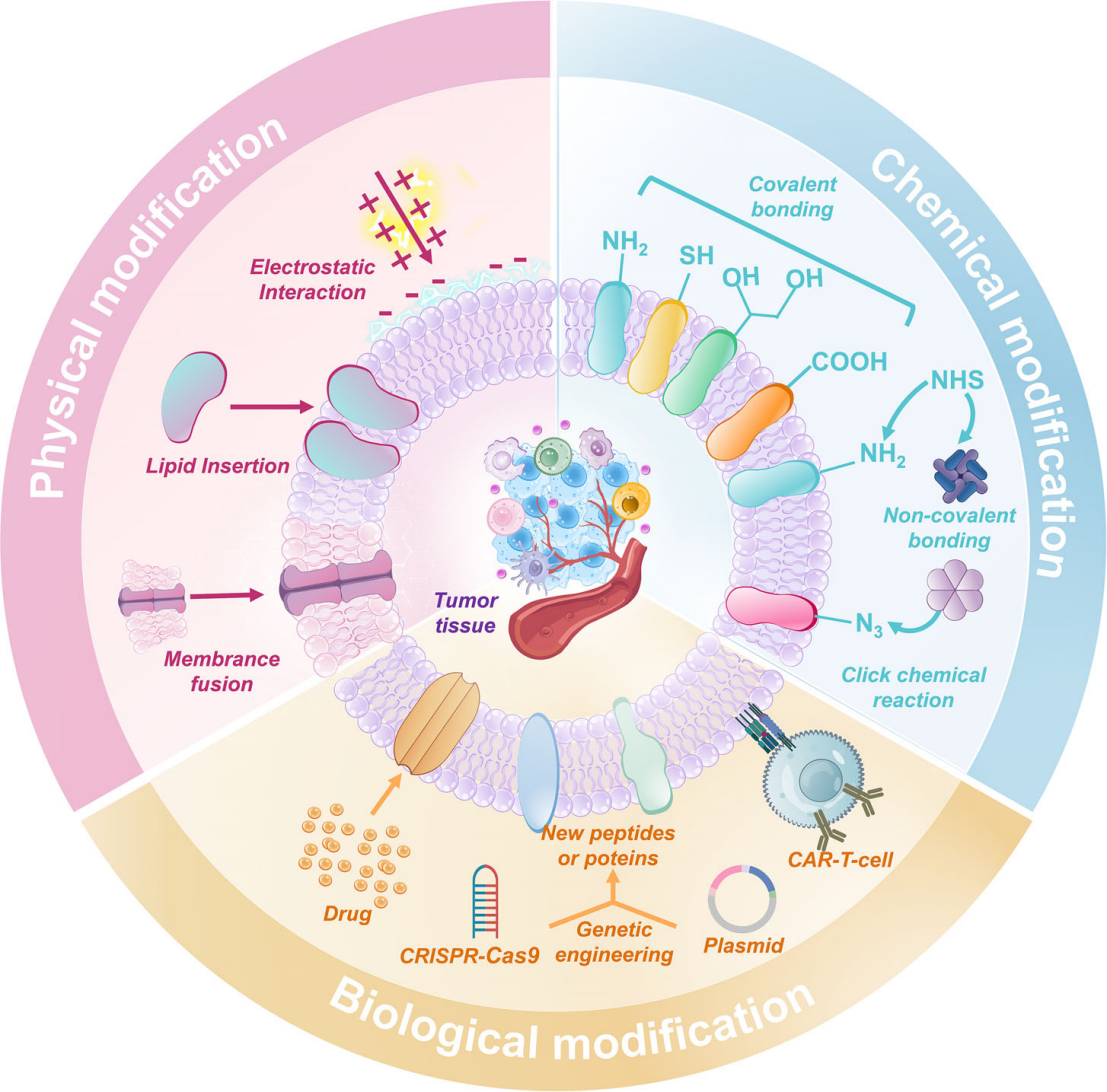
Engineering strategies for functional modification in biomembrane nanostructure.

With more and more studies exploring biomembrane nanostructures, the burgeoning demand for more specialized functionalities of biomembrane nanostructures is exceeding their natural properties. A growing number of researchers have engineered biomembrane nanostructures by reconstructing their fundamental constituent units to fine‐tune their biofunctions and enhance therapeutic efficacy.^[^ ^42^ ^]^ Mainstream strategies for functional refinement in engineering biomembrane encompass chemical modification, physical modification, and biological modification (**Figure** **2**).

#### 2.3.1 Chemical Modification

Biomembranes derived from CMs and EVs are mainly composed of phospholipid layers, polysaccharides, and functional membrane proteins. There are certain differences in the composition of biomembrane from different species sources, but they all provide a series of surface properties and functional groups. Functional therapeutic molecules could be modified the surface of biomembrane through chemical reaction.^[^ ^43^ ^]^

**Figure 3:**
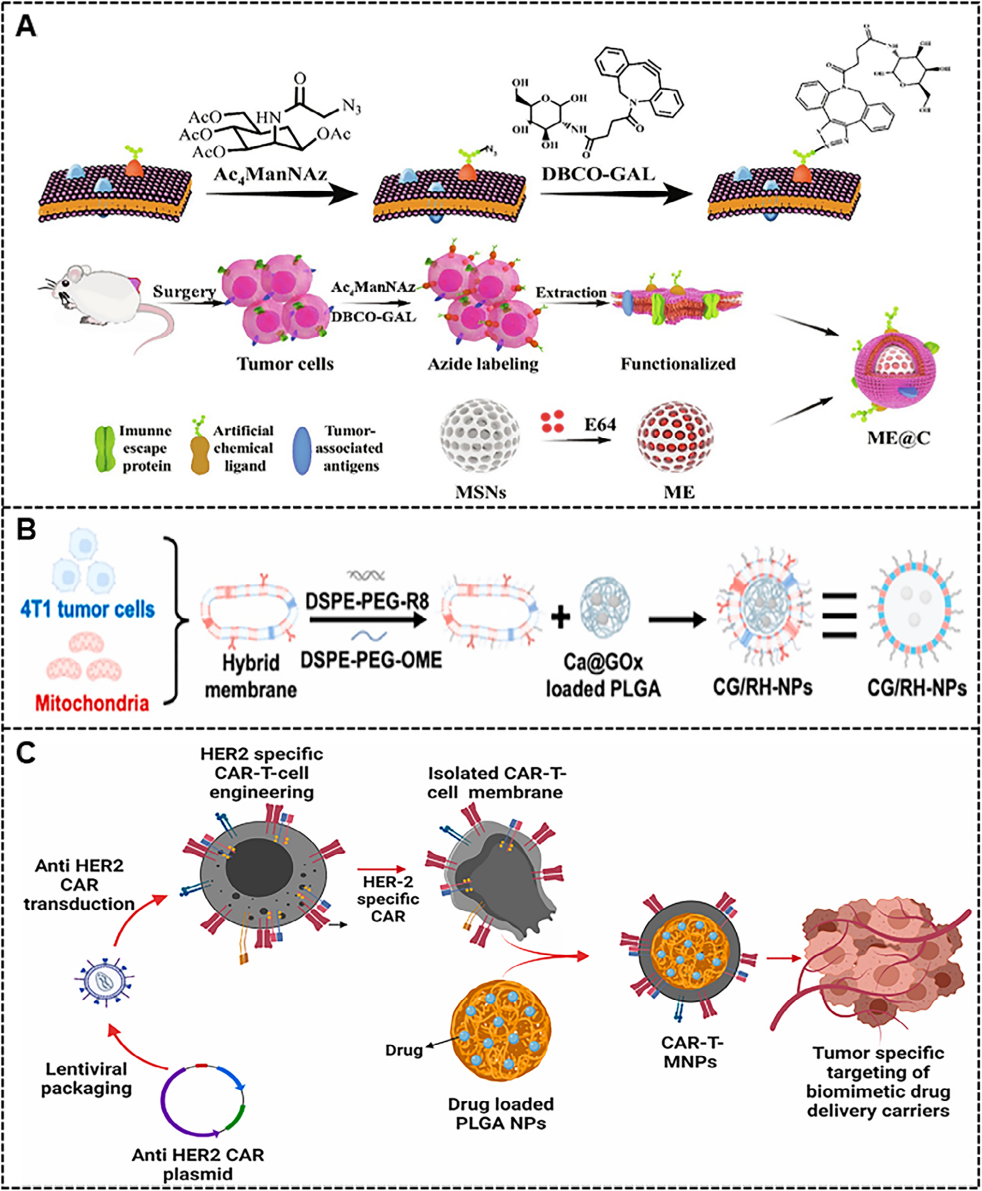
(A) Schematic illustration of azide labeling on CM through metabolic glycoengineering and following bioorthogonal labeling of GAL ligand, as well as the preparation process of modified CMs camouflaged mesoporous silica nanoparticle. Reproduced with permission.^[^ ^49^ ^]^ Copyright 2024, AAAS. (B) Visualization of biomimetic nanodrug coated with 4T1 tumor cell and mitochondrial hybrid membrane. Reproduced with permission.^[^ ^52^ ^]^ Copyright 2023, Elsevier B.V. (C) Description of the gene‐edited CAR‐T CM for targeted lung cancer therapy. Reproduced with permission.^[^ ^56^ ^]^ Copyright 2024, Elsevier B.V.

The membrane proteins on the surface of biomembrane contain numerous reactive functional groups, such as vicinal diol group, carboxyl group, sulfhydryl group, and amino group, which could be covalently bonded to external functional groups or bioactive molecules in a mild and efficient way.^[^ ^44^, ^45^, ^46^ ^]^ Commonly, the amino group from biomembrane could be chemically conjugated with the carboxyl group of the targeted bioactive molecules via 1‐ethyl‐3‐(3‐dimethyl aminopropyl)‐mediated amidation reaction.^[^ ^47^ ^]^ Non‐covalent binding strategies, like the avidin‐biotin interaction, also have emerged as powerful tools for chemical functionalization, enhancing specificity and delivery efficiency of therapeutic payloads. For example, Ding et al. modified a Y‐shaped targeting ligand that consists of p‐hydroxybenzoic acid and stabilized peptide onto red blood CMs by the mild avidin‐biotin interaction, achieving excellent barrier‐crossing ability for anti‐glioma therapy.^[^ ^48^ ^]^ Compared with traditional coupling reactions, bio‐orthogonal reactions could perform chemical modification in living cells without interfering with biochemical reactions. Particularly, the copper‐free click chemistry coupling method, as an efficient and precise reaction pathway, could realize biomembrane functionalization by the reaction process in which a compound with a cyclooctyne group undergoes a cycloaddition with an azide to form a triazole ring structure. Qiao and co‐workers first used tetraacetylated N‐azidoacetylmannosamine to integrate azide group on tumor CMs through metabolic glycoengineering, then dibenzocyclooctyne modified artificial ligand d‐galactosamine hydrochloride (DBCO‐GAL) was applied to paired with azide group introduced on the surface of CMs by copper‐free click chemistry, finally, artificial ligand‐modified tumor CMs was employed to camouflage mesoporous silica nanoparticles for cancer immunotherapy (**Figure** **3**A).^[^ ^49^ ^]^ Given that the functional reactive sites are located on the membrane proteins, increasing the number of modification sites will affect the biological function of membrane proteins, and excessive modification may cause the biomembrane to lose its function or undergo denaturation and aggregation. Therefore, attention should be paid to the number of modification sites to ensure the structural and functional integrity of the biomembrane.

#### 2.3.2 Physical Modification

Physical engineering modifies the surface of biomembrane mainly based on weak interaction forces, including electrostatic interaction, hydrophobic interaction, and so on. This modification process could better preserve the initial properties of biomembrane to the fullest extent with simple operation and broad applicability.

Anionic groups (e.g., carboxyl, phosphate, or sulfate groups) on the surface of biomembrane are primarily responsible for the negative charge of biomembrane. Various positively charged molecules (such as certain cationic polymers, cationic domains of proteins, etc.) could bind to the biomembrane surface through electrostatic interaction, relying on the Coulomb force between the charges.^[^ ^50^ ^]^ The amphipathic phospholipid structure of biomembrane allows functional ligands (from small molecules to large biomacromolecules) with lipophilic side chains to spontaneously insert into biomembrane through hydrophobic interaction. Sun et al. engineered OMVs from *Escherichia coli* by anchoring with Fe^2+^ under electrostatic interaction and decorating with tumor targeting 1,2‐Distearoyl‐sn‐glycero‐3‐phosphoethanolamine (DSPE)‐Polyethylene glycol (PEG)‐Folic acid (FA) under hydrophobic interaction to achieve enhanced ferroptosis antitumor immunotherapy.^[^ ^51^ ^]^ Additionally, biomembrane hybridization, a forefront technique in nanomedicine, fuses membranes originating from diverse sources to form hybrids possessing synergistically integrated functionalities through ultrasonication and coextrusion with the polycarbonate membrane. The extent to which specific functionalities could be reproduced is determined by the proportion of membranes involved. Deng et al. creatively fused the homologous 4T1 tumor CMs and corresponding mitochondrial membrane in a 1:1 mass proportion (Figure 3B), they proved that the hybrid 4T1 tumor cell‐mitochondrial membrane retained the homing effects of 4T1 tumor CMs and the targeting ability of subcellular membrane, and the hybrid membrane group exhibited greater therapeutic efficiency compared to the single membrane group.^[^ ^52^ ^]^ This innovative strategy motivates researchers to explore various combinations of biomembrane to achieve broader biological capabilities of CMs and more preferable therapeutics.

#### 2.3.3 Biological Modification

Biological modification permits the accurate modification of biomembrane without disturbing its native properties, and could avoid certain molecules that might interfere with each other and trigger immune responses. Drug stimulation and genetic engineering are commonly employed in biological modification to manipulate cellular behavior by regulating the expression of specific molecules on the surface of biomembrane.^[^ ^53^ ^]^

As a vital component of mammalian CMs, cholesterol plays an important role in maintaining the fluidity and stability of CMs. Li et al. engineered the cholesterol‐reduced T lymphocyte CMs by treating cells with (2‐hydroxypropyl)‐β‐cyclodextrin, and confirmed that it could effectively reduce the adsorption of complement proteins in the blood on the surface of biomembrane‐camouflaged nanoparticles, thereby decreasing the phagocytosis of core nanodrug by monocytes, while maintaining its tumor‐targeting ability.^[^ ^54^ ^]^ Genetic engineering, as a robust and highly versatile method, could precisely alter the expression of specific genes on the biomembrane, thereby achieving regulation of biomembrane structure and function. Techniques like virus transfection and CRISPR allow for the fabrication of genetically modified membranes, providing a cost‐efficient option for large‐scale production. Zhang et al. constructed 4T1 tumor CMs with highly expressed CD47 by gene transfection utilizing lentivirus plasmid in 4T1 cells, thus blocking the phagocytosis by macrophages and enhancing homologous targeting capacities by tumor cells through the combination of CD47 with signal regulatory protein alpha.^[^ ^55^ ^]^ Particularly, the gene‐edited chimeric antigen receptor (CAR) T‐CMs have been designed to express targeting molecule or single‐chain variable fragment (Figure 3C), paving the path for tumor‐targeted therapies.^[^ ^56^ ^]^

Overall, biomembrane engineering creates opportunities for the development of next‐generation nanomedicines with customized functions that go beyond natural limitations. Meanwhile, appropriate modification methods should be selected according to the application scenarios and modification purposes, or different modification methods can be combined to achieve the optimal modification efficiency.

## 3 Mammalian Cells‐Derived Biomembrane Nanostructures for Cancer Therapy

Cells in mammals are constantly interacting with each other through CMs or EVs. Every cell type possesses its own distinct components, characteristics, and functions, which provide motivation for scientists to customize antitumor nanodrugs (summarized in **Table** **2**).

**Table 2:** Examples of mammalian‐derived nanostructures in cancer therapy.

Source cells	Characteristics	Engineering strategies	Nanostructures	Tumor models	Refs.
RBC	Long circulation	Coextrusion	PFC@PLGA‐RBCM	4T1 breast tumor	[60]
	Simple structure	Sonication	FTP@RBCM	Hepa1‐6 tumor	[61]
		Coextrusion, lipid‐insertion	NTA630‐NCs‐RBCM‐T	HepG2 tumor	[62]
Platelet	Tumor targeting	Coextrusion	PLT‐Fe_3_O_4_	MCF‐7 breast tumor	[63]
	Simple structure	Sonication	PNP‐R848	MC38 colorectal tumor, 4T1 breast tumor	[156]
		Membrane fusion	DOX@PLT‐IL‐15	B16‐F10 melanoma	[157]
Macrophage	Antitumor properties	Coextrusion, drug stimulation	mSLP	4T1 breast tumor	[67]
	Intercellular adhesion	Coextrusion, gene transfection	PD‐1‐MM@PLGA/RAPA	Glioblastoma tumor	[158]
Neutrophil	Tumor targeting	Sonication	Neutrosome(L)‐cisplatin	A549 lung tumor	[68]
	Infammation targeting	Membrane fusion, electrostatic interaction	RNP@PNM	4T1 breast tumor	[69]
DC	Antigen presentation	Coextrusion	aDCM@PLGA/RAPA	Glioblastoma tumor	[70]
	Co‐stimulator	Click chemistry	DC‐N_3_‐IL15	E.G7‐OVA lymphoma	[71]
Lymphocyte	Tumor targeting	Sonication	TCM@PLGA	B16‐F10 melanoma	[74]
	Extravasation from blood vessels	Coextrusion, gene transfection, covalent binding	ORY‐BSA‐PD1‐M70	4T1 breast tumor	[73]
NK	Tumor targeting	Coextrusion	TCPP‐PLGA‐NKCM	4T1 breast tumor	[77]
	Tumor lysis	Coextrusion, lipid‐insertion	R‐NKm@NPs	Glioblastoma	[159]
Stem cell	Tumor homing	Coextrusion, lipid‐insertion	HMnO_2_‐MSC‐TAT@PTX	A549 lung tumor	[79]
	Long circulation				
		Coextrusion, drug stimulation	MOF@DPSCM	Oral squamous tumor	[80]
Cancer cell	Homologous targeting	Coextrusion, click chemistry	MSN‐ E64@CM	B16‐F10 melanoma,	[82]
	Antitumor vaccines			4T1 breast tumor	
		Gene transfection, drug stimulation, coextrusion	PEI25k/CpG‐NPs@CRT/CD47KO	B16‐F10 melanoma	[84]
		Coextrusion	AgPP@PLGA@M	4T1 breast tumor	[83]

### 3.1 Engineering CMs‐Derived Nanodrugs for Cancer Therapy

A range of natural CMs (including blood cells, immune cells, stem cells, cancer cells, etc) are utilized to camouflage synthetic nanomaterials. Depending on the type of CMs, the corresponding CMs‐derived nanodrugs have different merits. In this section, we delved into the significant advances in CMs‐mediated nanodrugs and discussed how they are conducive to optimizing cancer therapy outcomes.

#### 3.1.1 Blood CMs for Achieving Prolonged Circulation

**Figure 4:**
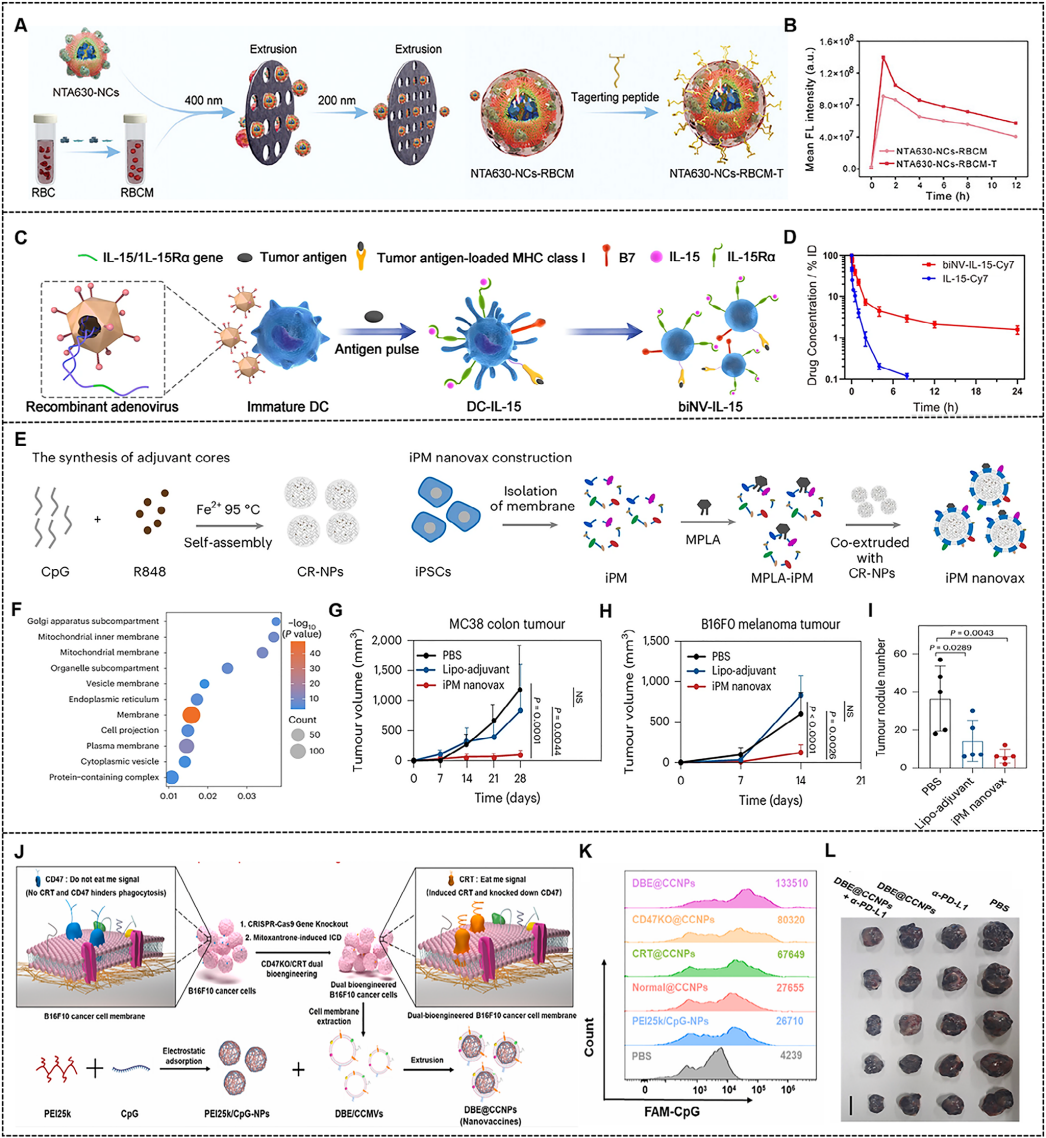
(A) Illustration of the construction of biomimetic nanodrug based on RBC membrane modified by targeting peptides. (B) Fluorescence quantification of biomimetic nanodrug based on RBC membrane with or without targeting peptides modification in nude mice. Reproduced with permission.^[^ ^62^ ^]^ Copyright 2025, Wiley‐VCH GmbH. (C) Production process of genetically engineered biNV‐IL‐15. (D) Pharmacokinetic profiles of Cy7‐labeled IL‐15 and biNV‐IL‐15 by evaluating the fluorescence of Cy7 in blood samples at different time points. Reproduced with permission.^[^ ^72^ ^]^ Copyright 2023, Springer Nature. (E) Schematic representation of the integrated nanovaccine consisting of iPM and commercial adjuvants. (F) Bubble plot presenting the significantly enriched gene ontology cellular components releated to the differentially highly expressed genes both in iPM and tumor CM. The average tumor volumes of (G) MC38 tumor model and (H) B16F0 tumor model during the preventive experiment. (I) Quantification of tumor nodules on lung tissues from 4T1 post‐operative lung metastasis of mice. Reproduced with permission.^[^ ^81^ ^]^ Copyright 2025, Springer Nature. (J) Illustration of the construction of CD47K/CRT dual bioengineering CM‐coated nanovaccine. (K) Endocytosis efficiency of different groups bioengineered nanovaccine by DCs. (L) Tumor images following the treatment experiments in B16F10 tumor models. Reproduced with permission.^[^ ^84^ ^]^ Copyright 2022, Elsevier B.V.

Prolonging the circulation duration of nanodrugs within the bloodstream is crucial for constructing outstanding delivery systems applicable in medical precision treatment. Red blood cells (RBCs), as oxygen delivery carriers, are the most representative and abundant form of blood cells with a half‐life of ≈120 days in the body. Self‐biomarkers expressed mainly on RBC membrane surface, such as CD47, an immunosuppressive protein, enable RBCs to avoid phagocytosis and systemic clearance by macrophages through interaction with signal regulatory protein alpha.^[^ ^57^ ^]^ Furthermore, RBCs are able to go through the sieving organs (liver and spleen) as well as narrow capillary networks. Inspired by these fascinating properties, such as long life‐span, limited clearance of the immune system, absence of nuclei and organelles. etc., RBCs membrane could be an attractive tool for nanodrugs to imitate the bionic feature in vivo. In 2011, Zhang and co‐workers first reported RBCs membrane‐camouflaged poly (lactic‐co‐glycolic acid) (PLGA) nanodrugs and achieved outstanding half‐life circulation reaching up to 39.6 h compared to conventional PEGylated nanodrugs with 15.8 h circulation time.^[^ ^58^ ^]^ It is widely acknowledged that the tumor microenvironment (TME) is distinguished by hypoxia, acidic pH, enzymes, and high interstitial fluid pressure, which lead to the restricted accumulation and distribution of nanodrugs or immune cells within tumor sites. Regulating TME is a valid strategy to boost antitumor efficacy. Inspired by oxygen‐carrying ability of RBCs, a series of oxygen‐generator materials (such as hemoglobin, perfluorocarbons, and catalase‐like nanozymes) have been developed to alleviate tumor hypoxia and elevate therapeutic outcomes.^[^ ^59^, ^60^ ^]^ Li et al. created artificial RBCs through RBC membrane‐coated Fe‐porphyrin‐based materials with catalase‐like activities to enhance multidimensional tumor catalytic therapy.^[^ ^61^ ^]^ Thereafter, RBC membrane has been extensively utilized to develop a biomimetic drug delivery system for antitumor therapy with prolonged circulation, including chemotherapy drugs, genes, enzymes, and theranostic nanomaterials (metal organic frameworks, polymers, mesoporous silica nanoparticles).^[^ ^24^ ^]^ Nevertheless, RBC membrane lacks specific active targeting ligands, restricting their efficacy in cancer treatment. Given this, lipid‐insertion technique was commonly employed to modify specific targeting molecules on RBC membrane through DSPE‐PEG lipid tethers, thereby enhancing the active targeting capacity of nanodrugs and their effective accumulation at tumor region (**Figure** **4**A,B).^[^ ^62^ ^]^

Platelets are another type of blood cells and also the smallest circulating blood constituent with 1–3 mm in diameter. They are derived from megakaryocytes in bone marrow and play an important role in the hemostasis process. Similar to RBCs, the collection and extraction of platelet membranes are relatively simple due to the anucleate structure. Platelet membrane surface is also equipped with “self‐recognized” proteins CD47, thus endowing platelet membrane‐derived nanodrugs with immune evasion ability and extended plasma half‐life.^[^ ^63^ ^]^ In recent years, it was reported that platelets have a crucial association with cancer metastatic progression based on the active interactions between platelets and tumor cells. For example, activated platelets were prone to cluster around circulating tumor cells, acting as a protective shield against the attacks of immune cells. In this context, Zhu et al. developed the platelet membrane‐hybridized liposomes (designated PM‐Lipo) to co‐deliver shikonin and quercetin for concurrently acting on platelets and cancer cells. Leveraging the specific affinity between P‐selectin molecules overexpressed on platelet membranes and CD44 receptors overexpressed on tumor cell membranes, PM‐Lipo could actively target the primary tumor site and exert its antitumor effect. Besides, PM‐Lipo is capable of binding to circulating tumor cells and diminishing their seeding within the premetastatic microenvironment, thereby inhibiting tumor metastasis.^[^ ^64^ ^]^

#### 3.1.2 Immune CMs for Modulating Immune Responses

Premalignant lesions and early‐stage tumor foci are typically infiltrated by various immune cells, including macrophages, neutrophils, dendritic cells (DCs), T lymphocytes, and natural killer cells (NKs). Numerous studies suggest that these immune cells play vital roles in the advancement and treatment of cancer. It has been confirmed that immune cells participate in activities such as remodeling TME, modulating immune responses, inhibiting tumor cell migration and invasion, through the complex signaling molecules on their membrane surfaces.^[^ ^65^ ^]^ Considering their special interactions with tumor cells and other immune cells, immune CMs show remarkable strengths in the treatment of cancer. Recently, numerous immune cell membrane‐derived nanodrugs have been fabricated for drug delivery and antitumor treatment.

Macrophages are the most abundantly accumulated innate immune cells within the TME through the macrophage‐tumor cell interactions, marked by their phagocytosis, antigen‐presenting capacity, and flexible phenotype. At different stages of the immune response, macrophages could be artificially divided into two subsets, M1 and M2. M1 macrophages could eliminate tumors by secreting proinflammatory cytokines and phagocytosis, and also present antigens to T cells to activate robust immune effects. However, M2 macrophages serve as anti‐inflammatory cells, having a significant impact on immune regulation and wound healing.^[^ ^66^ ^]^ Hence, membrane materials originating from diverse subsets of immune cells might endow distinct properties, or even entirely opposite impacts. Ying et al. first induced RAW264.7 macrophages to differentiate toward M1 phenotype using lipopolysaccharide (LPS). Subsequently, they extracted M1 macrophages membranes and coated them onto lipid nanodrugs. It was confirmed that there was specific protein expression of integrin (α4, β1) and vascular cell adhesion molecule‐1 on the prepared biomimetic nanodrugs, which enhanced nanodrugs' adhesion to tumor cells, improved systemic circulation, and strengthened tropism toward tumor lesions.^[^ ^67^ ^]^ Neutrophils are the most abundant innate immune cells in the human circulatory system and are also another kind of phagocyte, which possess the remarkable chemotactic property that enables them to migrate in a deformable manner to the inflammatory region around the tumor. Enlightened by this, a series of nanodrugs simulating neutrophils have been developed to inhibit tumors and mitigate metastasis by targeting circulating tumor cells within the bloodstream and suppressing the formation of premetastatic niches based on adhesion factors and ligands.^[^ ^68^, ^69^ ^]^

DCs are a specialized class of immune cells known for their capacity to capture and present antigens to other immune cells, thereby provoking specific immune responses directed at particular targets. Several specific molecules on the DCs' membrane surface and molecular interactions proved to be crucial for molding their immunological effects. For instance, major histocompatibility complex (MHC) molecules allow DCs membrane camouflaged nanodrugs to present antigens to T cells, thus triggering targeted immune responses; DCs membrane‐derived nanodrugs that preserve costimulatory molecules (CD80 and CD86) are capable of effectively activating T cells, affecting the intensity and feature of the subsequent immune response; DCs membrane‐derived nanodrugs could effectively increase the immunogenicity of related antigens and regulate the immune response through toll‐like receptors that are abundantly present on DCs membrane.^[^ ^70^, ^71^ ^]^ Wang et al. elaborately designed a biomimetic nanovaccine capable of delivering cytokine, which consisted of membrane vesicles, obtained from genetically engineered DCs with IL‐15/IL‐15 receptor α expression, tumor‐associated antigenic and costimulatory molecules (Figure 4C). Multiple tumor models (4T1, CT26, and B16F10‐OVA) demonstrated that biomimetic nanovaccine extended the cytokine's blood circulation with a half‐life 8.2‐fold longer than that of free IL‐15 and triggered broad‐spectrum antigen‐specific T cell responses while minimizing systemic adverse effects (Figure 4D).^[^ ^72^ ^]^

It has also been reported that immune cell membrane possesses an efficient antitumor immunotherapeutic function. Currently, T lymphocytes have grown to be increasingly significant mediators in antitumor immunity. Due to the abundance of proteins that can associate with tumor cells, like programmed death 1 (PD‐1) and adhesins LFA‐1, as well as inflammatory tendencies on T lymphocyte membrane, numerous investigations have employed T lymphocytes' membrane to conceal nanodrugs for cancer treatment via active targeting or immunological checkpoint strategies.^[^ ^73^ ^]^ Kang et al. wrapped T lymphocyte membrane derived from the EL4 cell line around poly(lactic‐co‐glycolic) acid loaded with dacarbazine to fabricate a biomimetic nanodrug named TCMNP for melanoma immunotherapy. Specifically, TCMNP could actively target tumors by adhesins LFA‐1, and TCMNP could directly eliminate cancer cells by releasing dacarbazine and triggering FasL‐mediated apoptosis. Additionally, TCMNP could eliminate immunosuppressive molecules like transforming growth factor‐β1 (TGF‐β1) and block immune checkpoints such as programmed death‐ligand 1 (PD‐L1) through TGF‐β1‐receptor proteins or PD‐1 on the plasma‐membrane proteins of TCMNP, respectively, thereby helping to restore the cytotoxic functions of cytotoxic T lymphocytes.^[^ ^74^ ^]^ This approach is more economical and less time‐consuming than adoptive T‐cell transfer therapy and exhibits lower systemic toxicity compared to immune checkpoint blockades based on superior tumor‐targeting efficacy and diverse treatment mechanisms. Similarly, NKs, as an essential component of the innate immune system, form the first line of defense against tumors and pathogens. NKs could efficiently target tumors through surface inhibitory receptors or activating receptors. For example, NKs are inclined to recognize the “missing self,” which means the absence of MHC class I that is typically presented on normal cells. Gene mutation and other factors lead to a reduction in the expression levels of MHC class I molecules on tumor cells. The inhibitory receptors, like killer cell immunoglobulin‐like receptors and natural killer group 2 A, will eradicate tumor cells with MHC class I negativity by regarding them as foreign entities. Additionally, NKs could also recognize stress‐induced ligands such as MHC class I chain‐related molecule A on the surface of tumor cells through their activating receptors. Moreover, distinct from T lymphocytes, NKs without antigen pre‐sensitization could lyse tumor cells through the exocytosis pathways of granzyme B and perforin, as well as provoke tumor necrosis via the Fas ligand or tumor necrosis factor‐related apoptosis‐inducing ligand.^[^ ^75^ ^]^ Considering this, NK‐based antitumor monotherapy or in combination with gene manipulation approaches like genetic modification and chimeric antigen receptor engineering have been broadly investigated because of their remarkable antitumor effectiveness in pre‐clinical and clinical studies.^[^ ^76^ ^]^ Deng et al. reported NK membrane‐cloaked photosensitizer nanoparticles to enhance photodynamic therapy (PDT) and augment antitumor immune efficiency. The acquired biomimetic nanodrugs selectively accumulated in tumors and induced or elicited the polarization of proinflammatory M1 macrophages by succeeding to the antigen spectrum of natural NK cells. Most significantly, this engineered NKs‐mimicking nanodrug not only eliminated primary tumors but also restrained the growth of distant tumors in the 4T1 tumor model.^[^ ^77^ ^]^

#### 3.1.3 Stem CMs for Improving Tumor Cell Tropism

It is well known that stem cells are capable of rolling and performing diapedesis within vascular endothelium and consequently arriving at tumor cells. Previous studies have shown that the interaction between certain signal molecules released by tumor cells and the corresponding receptors on stem CMs could initiate the homing behavior of stem cells. Moreover, stem cells have inherent tropisms for multiple types of tumors, such as breast cancer, colorectal cancer, and ovarian cancer.^[^ ^78^ ^]^ Furthermore, stem CMs could also diminish the clearance of the reticuloendothelial system (RES), making it possible to use stem cells originating from other species for non‐specific tumor therapy.

Mesenchymal stem cells (MSCs), as a class of widely‐sourced pluripotent stem cells with the abilities of low immunogenicity, migration, and homing to damaged tissues, paracrine effects, and immune regulation, have been studied extensively for antitumor therapy. These distinctive properties of MSCs are primarily credited to the diverse receptors on the membrane of MSCs, including growth factor receptors, cytokine receptors, chemokine receptors, cell‐matrix receptors, and cell‐cell interaction receptors. Inspired by this, MSCs membrane‐camouflaged nanodrugs present great potential application in antitumor treatments.^[^ ^79^ ^]^ Sun et al. constructed the biomimetic nanodrugs by coating dental pulp MSCs' membrane expressing CXCL8 receptor, CXCR2 on the metal‐organic framework loaded with doxorubicin (DOX) to treat oral squamous cell carcinoma. Oral squamous cell carcinoma usually secretes chemokine CXCL8 to attract dental pulp MSCs; the presence of MSCs' membrane enables biomimetic nanodrugs to accumulate in oral squamous cell carcinoma in vitro and in vivo via CXCL8/CXCR2 signaling pathway.^[^ ^80^ ^]^ It is well known that cells obtain a series of functional capabilities during the carcinogenic transformation; these alterations involve maintaining proliferative signaling, facilitating replicative immortality, counteracting cell death, and evading immune destruction. Crucially, induced pluripotent stem cells (iPSCs) display high similarities with tumor cells concerning the fundamental cancer‐shared characteristics, such as maintaining proliferative signaling and facilitating replicative immortality, which might be due to the reprogramming of iPSCs partly representing the oncogenic transformation of somatic cells. Li et al. fabricated a nanovaccine known as iPM nanovax composed of self‐assembled commercial adjuvant encapsulated in iPSCs‐derived membrane (Figure 4E). iPM nanovax was highly capable of boosting innate immunity and triggering B‐cell and T‐cell responses through epitopes from numerous antigens common to both tumor CMs and iPSCs‐derived membrane (Figure 4F), and effectively impeded the advancement of melanoma, colon cancer, breast cancer as well as post‐operative lung metastases (Figure 4G,I).^[^ ^81^ ^]^ This groundbreaking achievement has provided new possibilities for the development of universal cancer vaccines and also pointed out the direction for future immunotherapy research.

#### 3.1.4 Cancer CMs for Self‐Targeting and Presenting Antigens

Apart from the conventional cells noted above, cancer cells also hold great appeal for designing biomimetic nanodrugs. Specifically, cancer cells gradually evolve the ability for homotypic cell adhesion and immune evasion, thus escaping clearance by the immune system and forming stable tumor foci within the body. The homotypic cell adhesion capacity of tumors originates from multifarious specific adhesion glycoproteins present on the surface of cancer CMs, such as N‐cadherins, lectins, integrins, and epithelial cell adhesion molecules, which mediate efficient self‐recognition and facilitate homing to homologous tumor regions. Additionally, cancer cells could express certain “marker of self” proteins (like CD44, CD47, and CD200) and immune checkpoints (PD‐L1), which assist in evading immune‐mediated tumor clearance. Motivated by these characteristics, diverse cancer CMs‐coated nanodrugs have been constructed for tumor self‐targeting and effective cancer treatment.^[^ ^82^ ^]^ Wei et al. designed zeolitic imidazolate framework‐8 wrapped around 4T1 breast cancer CMs and demonstrated that the cellular uptake of 4T1 cells was greater than that of MDA‐MB‐231 cells and RAW 264.7 cells, which confirmed that proteins on the tumor CM contribute to homotypic aggregation and decrease macrophage internalization, thereby reducing clearance by the RES.^[^ ^83^ ^]^

In addition to tumor‐targeted drug delivery mentioned above, cancer CM‐coated nanodrugs could also be utilized to develop an innovative bio‐synthetic nanovaccine. Membrane‐attached tumor‐associated antigens rendered cancer CMs an effective option as an antigen resource to enhance antigen presentation and activate the immune response in vivo. Consequently, significant attempts have been directed toward using cancer CMs coated nanodrugs for targeted immunotherapy. Fang et al. wrapped PLGA in B16‐F10 mouse melanoma cell‐derived membrane and subsequently incorporated an immunological adjuvant monophosphoryl lipid A, the resulting nanodrugs could be efficiently transported to professional antigen‐presenting cells (APCs) for stimulating DCs maturation and downstream antitumor immune activation. Generally, cancer cells avoid immune surveillance by overexpressing immunosuppressive molecules that decrease their immunogenicity.^[^ ^26^ ^]^ Hence, enhancing the recognition and uptake of entire tumor antigens by APCs is a critical challenge for their utilization as tumor vaccines. Liu et al. first prepared CD47K/CRT dual bioengineered B1610 cancer cells by using gene editing technology and inducing immunogenic cell death (ICD) with mitoxantrone dihydrochloride, which ingeniously altered the biological signals on the surface of tumor CMs. Then the obtained CMs were wrapped around nanocore that was formed with hyperbranched PEI25k and cytosine‐phosphate‐guanine (CpG) to construct CD47K/CRT dual bioengineered nanovaccine (Figure 4J). This nanovaccine could facilitate the endocytosis of tumor antigens and CpG within DCs (Figure 4K) and promote their maturation as well as antigen cross‐presentation, and subsequently trigger tumor‐specific effector CD8^+^ T cells, further combined with anti‐PD‐L1 to inhibit tumor growth and invasion in melanoma mice (Figure 4L).^[^ ^84^ ^]^ Previous studies have demonstrated that senescent cells have the ability to convey antigens and activate APCs; senescent cancer cells achieve better performance compared to their pristine equivalents by recruiting innate and adaptive immune cells to assist in tumor regression. Yang et al. induced senescent melanoma B16‐OVA cells by incubating with a low dose of DOX, and engineered membranes were spread onto CpG‐loaded nanoadjuvant to generate a biomimetic nanovaccine. The results showed that nanovaccines constructed from the membranes of senescent cancer cells have greater efficacy in activating APCs and triggering antitumor protection under prophylactic circumstances compared to with nanovaccines sourced from cancer cells in a senescent state or undergoing ICD.^[^ ^85^ ^]^ This strategy helps to enhance the immunogenicity of vaccines and overcome the potential risks in the application of senescent tumor cell vaccines.

### 3.2 Engineering EVs‐Derived Nanodrugs for Cancer Therapy

EVs, serving as means of intercellular communication, have been proven to play a crucial role in regulating cell migration, cell apoptosis, and signal transduction. This capability brings tremendous promise for cancer treatment. Studies demonstrate that diverse types of cells excrete EVs into their surrounding environment. Among them, the most commonly applied EVs are produced by immune cells, stem cells, and cancer cells. Owing to their natural source, EVs possess characteristics such as excellent biocompatibility, low immunogenicity, and boundaries‐crossing capacity.^[^ ^39^ ^]^ In this section, we highlight the biological properties of EVs from various cells and explored the considerable developments in EVs‐mediated nanodrugs for cancer treatment.

#### 3.2.1 Immune Cell‐Derived EVs for Modulating Cancer‐Immunity Cycle

Immune cell‐derived EVs are attracting notice in terms of drug delivery applications. They inherit traits from their parental cells and actively engage in both innate and adaptive immune responses. Immune cells are categorized into two groups: innate immune cells (macrophages, NKs, DCs, neutrophils) and adaptive immune cells (T cells, B cells). Innate immune cells promptly react to pathogens, triggering nonspecific immune responses, and have the ability to clear cancer cells. Adaptive immune cells could evoke specific immune responses, identifying specific antigens associated with tumors, and could precisely destroy cancer cells. Due to the significant functional disparities among diverse immune cells, EVs from different sources exhibit distinct biological properties.^[^ ^86^ ^]^

**Figure 5:**
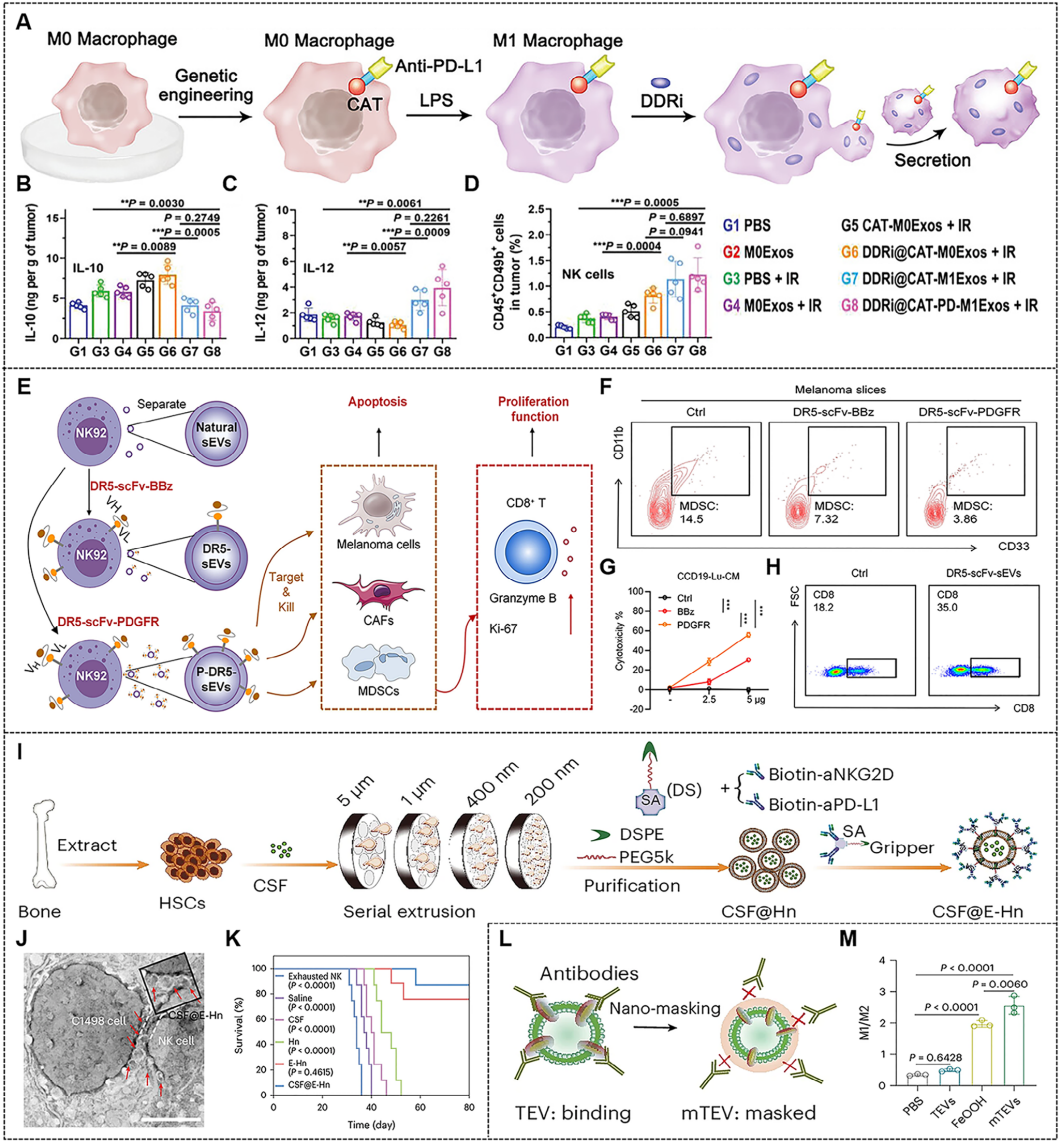
(A) Schematic illustration of designing engineered M1 macrophage‐derived EVs. (B) IL‐10 and (C) IL‐12 expression level in tumor was detected by ELISA. (D) The percentage of CD45^+^CD49b^+^ (NK cells) in tumor was analyzed by flow cytometry. Reproduced with permission.^[^ ^87^ ^]^ Copyright 2022, Wiley‐VCH GmbH. (E) Schematic diagram of NKs‐derived EVs platform for cancer therapy. (F) Flow cytometry analysis of CD33^+^CD11b^+^ MDSCs in patient‐derived melanoma tissue slices treated with NKs‐derived EVs. (G) CAF (induced by CCD19‐Lu cells) killing assay through the treatment of NKs‐derived EVs. (H) Flow cytometry analysis of CD8^+^ T cells among CD3^+^ T cells in patient‐derived melanoma tissue slices treated with NKs‐derived EVs. Reproduced with permission.^[^ ^88^ ^]^ Copyright 2025, AAAS. (I) Diagram of functional HSCs‐derived EVs. (J) Transmission electron microscopy image displays that functional HSCs‐derived EVs is approaching C1498 and NK cells. (K) Survival curves of leukemia‐bearing mice treated by different formulations. Reproduced with permission.^[^ ^98^ ^]^ Copyright 2024, Springer Nature. (L) Schematic illustration shows the mechanism by iron oxide hydroxide layer blocks the immune‐evasive EV surfaces. (M) Relative M1/M2 ratio in tumor sections after intratumoural injection of different formulations. Reproduced with permission.^[^ ^103^ ^]^ Copyright 2025, Springer Nature.

Innate immune macrophage‐derived EVs are capable of efficiently participating in cancer immune regulation to combat cancer. EVs from macrophages exhibit multifarious functions hinging on the diverse phenotypes of their parent cells. Ma et al. constructed engineered M1 macrophage‐derived EVs expressing anti‐PD‐L1 and catalases on the surface for delivering DNA damage repair inhibitor (DDRi). The cytomembrane of RAW 264.7 cells was engineered by lentiviral transfection of exogenous protein catalase‐TMR‐anti‐PD‐L1‐c‐myc, and then genetically engineered RAW 264.7 cells were polarized into M1 phenotypes by LPS. Subsequently, engineered M1 macrophage‐derived EVs were acquired from genetically engineered RAW 264.7 cells following the uptake of DDRi (**Figure** **5**A). The catalase expressed in engineered M1 macrophage‐derived EVs resulted in the alleviation of tumor hypoxia, and the DDRi loaded in engineered EVs led to the suppression of DNA damage repair. Moreover, the natural features of M1 macrophage‐derived EVs could turn M2 macrophages into M1 phenotypes, thereby encouraging macrophages to release cytokines that are helpful for the antitumor immune system (Figure 5B,C). Eventually, the suppressive immune microenvironment of the tumor was reversed based on the natural properties of M1 macrophage‐derived EVs and the anti‐PD‐L1 antibody (Figure 5D).^[^ ^87^ ^]^ NKs could non‐specifically kill abnormal cells without antigen pre‐stimulation. EVs derived from NKs also exert cytotoxic effects on tumor cells, like melanoma and breast cancer in vivo. Guo et al. developed an engineered NKs‐derived EVs platform with high‐efficiency expression of death receptor 5 (DR5) through plasmid transfection (Figure 5E), and the platform could inhibit tumor growth and restore the normal functions of other immune cells by specifically targeting DR5^+^ tumor cells, myeloid‐derived suppressor cells (MDSCs), and cancer‐associated fibroblasts (CAFs) (Figure 5F–H).^[^ ^88^ ^]^ EVs derived from DCs could modulate immune functions, facilitate immune‐cell‐driven tumor suppression, and have been utilized for stimulating antitumor immune responses in clinical trials, and have a superiority over DCs‐based immunotherapies. Recent research has demonstrated that EVs obtained from DCs maintain the initial conformation, structural soundness, and immune functionality of their parent DCs membrane. Xia et al. fabricated cationic nanoparticles co‐encapsulated with ovalbumin and adjuvant cdGMP and swallowed them within EVs derived from ovalbumin‐stimulated DCs, namely swallowing nanovaccines. Compared to cationic nanoparticles alone, swallowing nanovaccines could augment the antigen load, further strengthen the immune response due to the existence of peptide‐MHC, and could home to lymph nodes and interact with DCs by means of their homotypic targeting characteristics.^[^ ^89^ ^]^ EVs derived from neutrophils are capable of triggering apoptosis in tumor cells but not in normal cells by transporting cytotoxic proteins such as FasL, granzyme, perforin, and initiating the caspase signaling pathway.^[^ ^90^ ^]^

Adaptive immune T lymphocytes‐mediated cytotoxicity against tumors is contingent upon the activation of T cells as well as their infiltration into tumors. T lymphocytes are categorized into two subsets, predominantly CD8^+^ cytotoxic T lymphocytes and CD4+ helper T cells. EVs derived from T cells are capable of arousing the activity of immune cells, incorporating FasL and granzymes, and facilitating T‐cell activation in the fight against cancer. Hong et al. constructed T lymphocyte‐derived nanovesicles with highly expressed PD‐1, TGF‐β receptor, which block PD‐L1 on cancer cells and scavenge TGF‐β in the immunosuppressive TME, thereby preventing cytotoxic‐T‐cell exhaustion, while the nanovesicles directly kill cancer cells by delivering granzyme B. Ultimately, the nanovesicles effectively inhibit the growth of tumors within syngeneic‐solid‐tumor‐implanting mice.^[^ ^91^ ^]^ CD4+ T lymphocytes, especially those stimulated by interleukin‐2, could derive EVs that augment the proliferation and activity of CD8^+^ T lymphocytes without influencing regulatory T cells via endogenous miRNAs, thus facilitating the antitumor response of CD8^+^ T lymphocytes.^[^ ^92^ ^]^ Specifically, CAR‐T cells have achieved success in treating blood cancer. EVs derived from CAR‐T cells also demonstrated great potential in cancer treatment. It has been reported that EVs derived from CAR‐T cells transport CAR and contain high concentrations of cytotoxic molecules, enabling them to markedly suppress tumor growth with better safety.^[^ ^93^ ^]^ B cell‐derived EVs could offer immunogenic stimulation by expressing several molecules like MHC II, LFA‐3 to assist in the activation of CD4+ T cells and also induce the responses of CD8^+^ T lymphocytes.^[^ ^94^ ^]^

#### 3.2.2 Stem Cell‐Derived EVs for Transmitting Antitumor Signals

Stem cell‐derived EVs are heterogeneous nanovesicles that mediate intercellular communication within TME by transferring metastasis‐related signaling molecules, including regulatory proteins and miRNAs, to the recipient cells such as cancer cells, immune cells, and cancer‐associated fibroblasts. Stem cell‐derived EVs could exhibit both pro‐tumorigenic and antitumorigenic effects, which are critically dependent on their carrying substances and tissue sources. For instance, bone marrow MSC‐derived EVs promote osteosarcoma progression via the specific lncRNA MALAT1, whereas adipose tissue MSC‐derived EVs activate the mTOR pathway to enhance hepatocellular carcinoma chemosensitivity.^[^ ^95^, ^96^ ^]^ These findings underscore the importance of tissue source selection and cargo characterization in harnessing MSC‐derived EVs for therapeutic applications. Therefore, prioritizing MSC‐derived EVs exhibiting inherent tumor‐suppressive activity is essential for optimizing therapeutic efficacy and minimizing potential oncogenic risks in drug delivery systems targeting malignancies.

Simultaneously, engineering stem cell‐derived EVs has received widespread attention owing to their therapeutic potential in regulating antitumor activity as well as repairing damaged tissues. Particularly, MSC‐derived EVs possess low immunogenicity, stable phenotype relative to their parental cells, and are economically efficient when manufactured in large amounts. Bagheria et al. encapsulated DOX into MSC‐derived EVs through electroporation and covalently decorated MUC1 aptamer to provide targeted drug delivery, which demonstrated increased tumor accumulation and accelerated liver clearance compared to free DOX.^[^ ^97^ ^]^ Zhang et al. loaded colony‐stimulating factor into haematopoietic stem cell (HSC)‐derived EVs with aPD‐L1 and aNKG2D to bring NKs and tumor cells in closer proximity, simultaneously activating endogenous NKs for specific tumor cell targeting and eradication within bone marrow TME (Figure 5I,J). This system demonstrated excellent therapeutic effects in mouse models of acute myeloid leukemia and multiple myeloma, and was able to prevent tumor recurrence in the long term (Figure 5K).^[^ ^98^ ^]^ More promisingly, MSCs‐functionalized nanodrugs could target cancer cells with unidentified surface target membrane antigen proteins, namely cancer cells lacking known clinically available receptors. And the unidentified antigen proteins on the cancer CM account for over 90% of the surface proteins. Park et al. developed a strategy focusing on educational stem cells to target and destroy malignant deep pancreatic tumor cells. Concretely, co‐culturing MSCs with pancreatic tumor cells could promote the increased expression of target receptors on MSCs' membrane aimed at tumor cells, such as C‐C chemokine receptor (CCR1, CCR2) and C‐X‐C chemokine receptor (CXCR4); RNA‐sequencing analysis showed that educational MSCs exhibited upregulated genes related to migration, inflammatory response, immune response, angiogenesis, growth factors, and surface proteins of pancreatic tumor cells compared with MSCs. Then separating EVs from educational MSCs with the integration with PLGA encapsulating DOX showed greater antitumor efficacy and targeting ability in patient‐derived xenograft mice by intraperitoneal injection compared with gemcitabine, an antitumor drug commonly used in the clinical treatment of pancreatic tumors.^[^ ^99^ ^]^ This study represented an innovative therapeutic approach for the management of intractable tumors.

#### 3.2.3 Cancer Cell‐Derived EVs for Regulating the TME

Cancer cells generate numerous homing‐competent EVs that express substantial amounts of MHC‐I and tumor markers presented on their surface, which are of great significance for cell reprogramming in the intricate TME. Previous studies demonstrated that cancer cell‐derived EVs possess excellent tropism, enabling them to effectively target the tumor cells themselves, thereby influencing tumor progression and TME. This inspired the utilization of cancer cell‐derived EVs as promising carriers for delivering antitumor drugs (such as small molecules, proteins, and nucleic acids).^[^ ^100^ ^]^ Peng et al. utilized EVs derived from 4T1 tumor cells to encapsulate anti‐STAT3 short interfering RNA and DOX for treating triple‐negative breast cancer. In vitro and in vivo experiments showed that the biomimetic nanodrugs accurately targeted tumor tissues, effectively downregulate the expression of STAT3, and synergistically induce tumor ICD through a synergistic chemo‐gene therapeutic approach, thus remodeling the immunosuppressive tumor TME and remarkably inhibiting the growth of primary tumors and distant metastases.^[^ ^101^ ^]^

Meanwhile, cancer cell‐derived EVs gained widespread attention in antitumor vaccine development by acting as neoantigen components and adjuvants due to their distinctive tumor‐specific proteins. Nevertheless, it is known that cancer cell‐derived EVs paradoxically exhibit pro‐tumorigenic properties such as enhancing metastatic potential and inducing therapeutic resistance, as well as inherent immunosuppressive characteristics such as impairing immune cells' activity, which hinders their application in antitumor therapies. To overcome these drawbacks, many studies prioritized engineering cancer cell‐derived EVs to preserve their advantages while eliminating undesirable pro‐tumorigenic characteristics. Han et al. proposed an innovative bioengineering strategy to reuse cancer cell‐derived EVs as personalized therapeutic vaccines by treating tumor cells with verteporfin to generate attenuated yet immunogenically potentiated EVs. This refined vaccine effectively attenuated the malignant phenotypes of cancer cell‐derived EVs by simultaneously suppressing Yes‐associated protein signaling and autophagic flux, while amplifying the immunogenicity through abundant antigens and adjuvants on autologous cancer cell‐derived EVs. In vivo experiments confirmed that the resulting engineered vaccine could inhibit tumor growth by prompting tumor‐specific immune responses and inducing long‐lasting immune memory in both prophylactic and recurrence vaccination models.^[^ ^102^ ^]^ The efficacy of personalized cancer immunotherapy relies on efficient tumor antigen presentation to antigen‐presenting cells. Although cancer cell‐derived EVs carry substantial tumor‐associated antigens, the anti‐phagocytic signals present on the surface of cancer cell‐derived EVs, such as CD47, lead to immune evasion of DCs and macrophages. Ding et al. covered the CD47 signals by depositing a thin film of iron oxide hydroxide on the surface of the isolated cancer cell‐derived EVs via a mild surface reaction while preserving their antigenic payload (Figure 5L). The nanocomposite coating overcame the prevalent phagocytosis resistance observed in patient‐derived EVs, concurrently inducing macrophage phenotype reprogramming in both animal tumor models and clinical malignant pleural effusion specimens (Figure 5M), resulting in significant tumor regression and metastasis suppression.^[^ ^103^ ^]^

## 4 Bacteria‐Derived Biomembrane Nanostructures for Cancer Therapy

Antitumor bacterial therapies possess advantages over conventional methods, such as the potential control after administration, the cytotoxicity toward multidrug‐resistant cancer cells, and the simplicity of design and modification. So far, several bacteria species‐based antitumor strategies have progressed to clinical trials for antitumor therapy, including attenuated *Salmonella typhimurium*, *Clostridium novyi*, *Escherichia coli*, *Listeria monocytogenes*, and *Bifidobacterium*.^[^ ^104^ ^]^ However, the clinical translation of these approaches continues to face significant barriers, particularly regarding infection‐related adverse effects. Achieving an optimal balance between antitumor efficacy and systemic safety represents a persistent challenge. Besides bacteria serving as therapeutic agents and tools for gene cloning, nanoscale bacteria‐derived membrane nanostructures, including BMs, OMVs, have been developed for drug delivery (summarized in **Table** **3**).^[^ ^105^ ^]^ Compared with natural bacteria, these bacteria‐derived membrane nanostructures are smaller and more stable, rendering them more appropriate for in vivo research.

**Table 3:** Examples of bacterial‐derived nanostructures employed in cancer therapy.

Types	Characteristics	Engineering strategies	Nanostructures	Tumor models	Refs.
BM	Immunogenic	Noncovalent binding	DOX‐ghosts	–	[107]
	Adjuvant	–	Bacterial ghosts	CT26 colorectal tumor	[108]
		Coextrusion	COF‐306@FM	4T1 breast tumor	[109]
		Coextrusion, lipid‐insertion	PC7A/CpG@BM‐Mal	NXS2 neuroblastoma, B78 melanoma	[106]
		Sonication	SM‐AuNRs	HCT‐116 colorectal tumor	[160]
		Coextrusion, click chemistry	VA‐SAM@BTO	CT26 colorectal tumor	[161]
OMV	Immune stimulation	Co‐incubation	DOX/Ce6‐OMVs@M	4T1 breast tumor	[112]
	Macrophage polarisation	Sonication	MPD@DMV	B16‐F10 melanoma	[162]
	Antigen presentation	Gene transformation	OMVs‐tumor antigens	B16‐F10 melanoma	[116]
		Gene knockout, sonication, lipid‐insertion	Angiopep‐2‐OMVs@PTP/siCd47‐aDOX	Glioblastoma	[154]
		Covalent binding, electroporation	mUNC2025@OMVs	B16‐F10 melanoma	[163]
		Gene transformation, covalent binding	OMV‐antigen‐αPD‐L1	MC38 colorectal tumor, Pa02 pancreatic tumor	[164]
		Membrane fusion	mTOMV	4T1 breast tumor, CT26 colorectal tumor	[118]

### 4.1 Engineering BMs‐Derived Nanodrugs for Cancer Therapy

**Figure 6:**
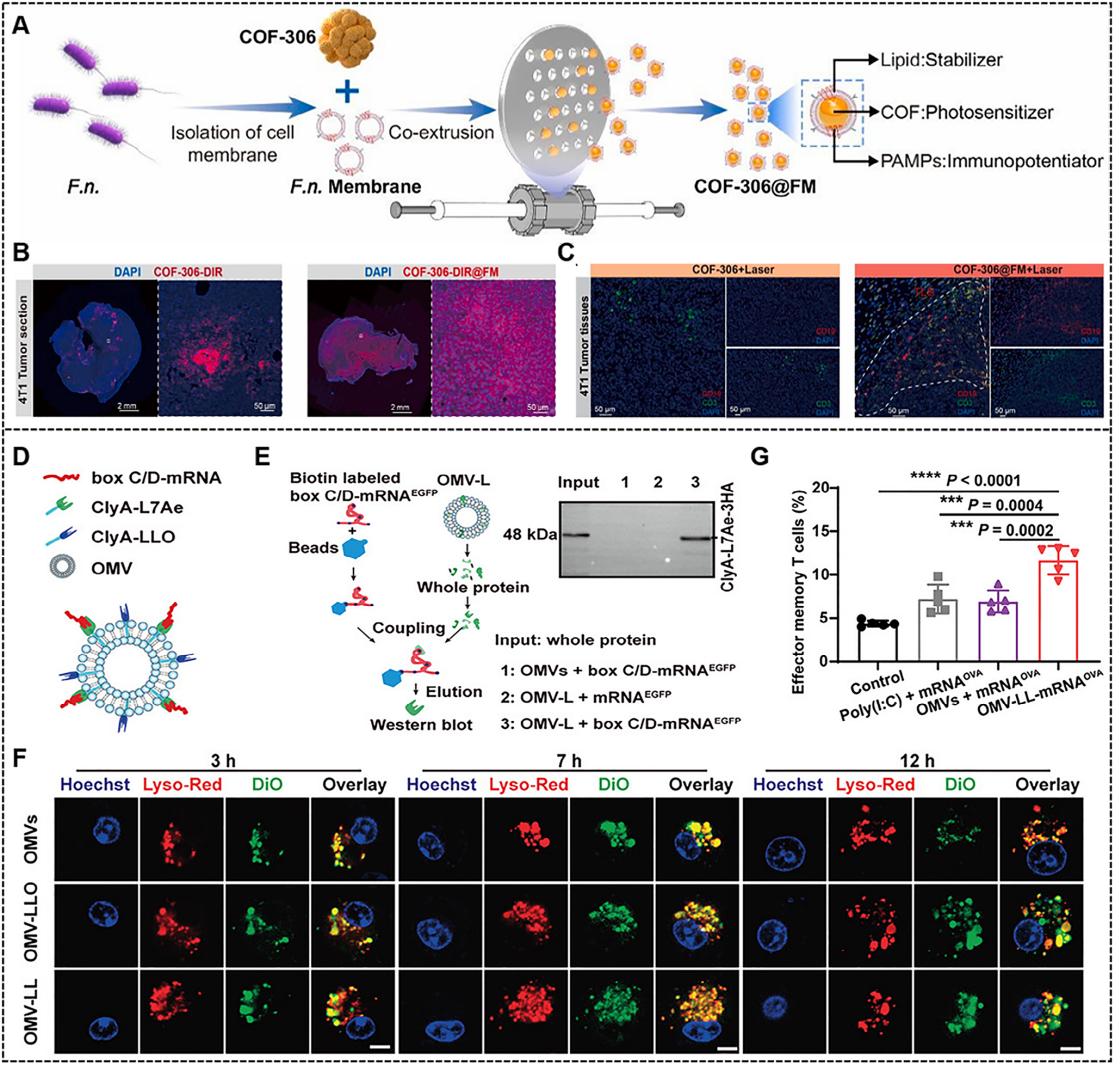
(A) Schematic representation of F.n. membrane‐cloaked COF‐306. (B) Distribution of COF‐306 and COF‐306@FM within 4T1 tumors following intratumoral injection for 24 h. (C) Multiplex immunohistochemical staining images of tertiar lymphoid structures within tumors after different treatment. Reproduced with permission.^[^ ^109^ ^]^ Copyright 2025, Elsevier B.V. (D) Schematic diagram of OMV‐based mRNA delivery system. (E) RNA pull‐down assay assessing the binding of OMV‐L and box C/D‐mRNA^EGFP^. (F) Intracellular kinetic level of OMV‐LL in DCs using confocal fluorescence microscopy. (G) Quantitative analysis of effector memory T cells (CD3^+^CD8^+^CD44^+^CD62L^–^) in splenocytes. Reproduced with permission.^[^ ^115^ ^]^ Copyright 2022, Wiley‐VCH GmbH.

BMs are bacterial empty envelopes generated by removing cytoplasm and nucleic acids, while eliminating the adverse toxicity of live bacteria, which could act as a pioneering delivery system to store certain therapeutic agents, including proteins, DNA, enzymes, and synthetic materials in their internal space for diverse biomedical applications. Furthermore, BMs are inherently immunogenic and exhibit intrinsic adjuvant properties attributed to the retention of diverse pathogen‐associated molecular patterns (PAMPs), including immunostimulatory molecules LPS, flagellar proteins, or peptidoglycan. These membrane molecules are pivotal in modulating the host immune system, endowing BMs with natural adjuvant activity and making them highly promising vaccine candidate materials.^[^ ^106^ ^]^ Wrapping BMs around therapeutic agents could replicate the process in which bacteria present natural antigens to the immune system. Gram‐negative bacteria, along with gram‐positive bacteria, could be employed to create biomimetic nanosystems, with functionalities predominantly stemming from the characteristics of the original bacterial species. The dense peptidoglycan layers of gram‐positive species raise the complexity in fabrication, whereas gram‐negative species could be engineered more flexibly due to thin peptidoglycan layers, thus becoming the focus of further research. It has been proven that BMs obtained from gram‐negative bacteria (*M. haemolytica* A23) could transport DOX to human colorectal adenocarcinoma cells, and the specific drug‐targeting properties of BMs have remarkably enhanced the antiproliferative ability.^[^ ^107^ ^]^ Groza et al. stated that the application of BMs could augment the oxaliplatin‐induced ICD, elicit long‐term antitumor memory effects and result in a strong synergistic antitumor effect against the CT26 allograft, evidencing the immune‐stimulatory capabilities of BMs.^[^ ^108^ ^]^ Yang et al. harnessed *Fusobacterium nucleatum* (*F.n*.), a prevailing tumor‐colonizing bacterium, to engineer a *F.n*. membrane‐cloaked covalent organic framework (COF‐306@FM) (**Figure** **6**A). The *F.n*. membrane attained dual functionality in this nanotherapeutic system. On the one hand, *F.n*. membrane significantly enhanced the endocytic internalization efficiency of COF‐306@FM while ensuring uniform distribution within tumor tissues (Figure 6B). On the other hand, *F.n*. membrane exhibited adjuvant‐like immunomodulatory properties, elevating the systemic antitumor responses by synergizing with COF‐mediated phototherapy (Figure 6C). Such dual mechanisms collectively established a self‐reinforcing therapeutic cycle and efficiently inhibited tumor metastasis and recurrence.^[^ ^109^ ^]^

### 4.2 Engineering OMV‐Derived Nanodrugs for Cancer Therapy

OMVs are spherical bilayer nanostructures with diameters ranging from 20 to 250 nm that are released from the bacterial outer membrane. The size effect endows OMVs with certain tumor‐targeting capabilities, and targeting modifications could further enhance the targeting specificity of OMVs to particular cells. More importantly, these biologically active nanovesicles inherently preserve multiple immunogenic antigens and PAMPs from the parental bacteria, making them valuable for antitumor therapy.^[^ ^110^ ^]^

In antitumor research, OMVs could serve not only as carriers for the delivery of antitumor drugs, but also as immunomodulators to reshape the TME and activate the body's immune response, thus synergizing with therapies such as chemotherapy or phototherapy to inhibit tumor growth. It was reported that applying Escherichia coli OMVs to coat β‐cyclodextrin‐modified gold nanoparticles (GNPs) and adamantane‐modified GNPs, respectively could trigger the selective uptake of GNPs by phagocytic immune cells and subsequently drive the supramolecular self‐assembly of GNPs by β‐cyclodextrin‐adamantane host‐guest interactions, proving the boosted targeting efficiency and treatment effects in melanoma tissues.^[^ ^111^ ^]^ Li et al. first confirmed that OMVs could function as immuno‐stimulating agents to polarize M2 macrophages into M1 phenotypes and then utilized macrophages as mediator to deliver OMVs loaded with the photosensitizer chlorin e6 and chemotherapeutic drug DOX, achieving complete inhibition of triple‐negative breast tumors in mice and successfully preventing the occurrence of tumor metastasis through combinational chemo‐/photodynamic/immunotherapy.^[^ ^112^ ^]^ Meanwhile, OMVs could recruit various immune cells, including NK cells and T cells, which not only caused the reversal of immune‐suppressive TME but also induced the production of interferon (IFN‐γ) within TME to mediate tumor apoptosis.^[^ ^113^ ^]^ Furthermore, OMVs themselves have a certain killing effect on tumors. The PAMPs enriched on the surface of OMVs could induce cell pyroptosis or apoptosis through receptor‐activated death pathways at the cellular level. LPS is one of the most abundant components of OMVs. Studies have demonstrated that utilizing OMVs as a natural vehicle to convey LPS to tumor cells could activate the noncanonical pyroptosis pathway for immunotherapy.^[^ ^114^ ^]^ Specifically, OMVs‐induced cell death is influenced by multiple factors, including the composition of OMVs, the relative concentrations of PAMPs, and the sensitivity of host cells.

In tumor vaccine research, engineered OMVs could not only rapidly capture a variety of tumor antigens, but also target lymph nodes and enhance the antigen uptake by DCs. Li et al. developed an OMVs‐based nanoplatform engineered as a novel mRNA delivery system through surface functionalization with RNA binding protein L7Ae and lysosomal escape protein listeriolysin O (designated OMV‐LL) (Figure 6D), OMV‐LL efficiently absorbed mRNA antigens labeled with box C/D sequences through the binding of L7Ae and then delivered them into DCs (Figure 6E), followed by the cross‐presentation through the endosomal escape mediated by listeriolysin O (Figure 6F). In preclinical evaluation, this tumor vaccine successfully achieved the inhibition of tumors and induced long‐term immune memory (Figure 6G).^[^ ^115^ ^]^ Additionally, loading foreign peptides onto/into OMVs is also an essential step in producing OMVs‐based tumor vaccines. Bioengineering methods enabled OMVs‐based nanoplatform to flexibly present multiple tumor antigens. Cheng et al. developed a flexible OMVs‐based vaccine through the specific presentation of antigens on the OMVs surface. Tumor‐associated antigens were anchored onto OMVs surface by fusing them with ClyA protein. Then, the antigen presentation process was optimized by utilizing Plug‐and‐Display system composed of tag/catcher protein pairs. Delivering a series of tumor antigens by OMVs‐based nanovaccine in a mouse tumor model achieved an outstanding antigen‐specific T lymphocyte‐mediated antitumor immune response. This bioengineered OMVs system is capable of simultaneously displaying multiple tumor antigens, which may be valuable for the development of personalized tumor vaccines that aim at intricate and heterogeneous tumor antigens.^[^ ^116^ ^]^ Simultaneously, OMVs could act as adjuvants to further boost the tumor‐specific immune response. Liu et al. intraperitoneally administered *E. coli*‐derived OMVs to mice one week before the tumor vaccine OVA administration. It has been found that these OMVs could activate the inflammasome signaling pathway and induce the secretion of interleukin‐1β, thereby increasing the production of antigen‐presenting cell progenitors, ultimately leading to an enhanced immune response and increased activation of tumor antigen‐specific T cells when antigens were delivered.^[^ ^117^ ^]^ Therefore, the OMVs trained immunity strategy holds promise as an approach to positively direct vaccine‐induced adaptive immune responses. Additionally, fusing OMVs with cancer CMs is able to establish an integrated system through spatial colocalization of tumor antigens and natural adjuvants, which leverages the complementary biological characteristics of both membrane sources, offering enhanced immunotherapy potential.^[^ ^118^ ^]^

## 5 Plant‐Derived Biomembrane Nanostructures for Cancer Therapy

Plant‐derived biomembrane nanostructures have demonstrated significant therapeutic potential in recent studies. Among these, thylakoid membranes (TKs), primarily extracted from spinach, are the most widely utilized due to their unique properties, including photosynthetic oxygen generation, photothermal conversion, PDT, and peroxidase‐like catalytic activity, and these functionalities collectively address tumor hypoxia and enable multimodal cancer therapeutics.^[^ ^119^ ^]^ Furthermore, with the advancements of nanoscience, there is an increasing focus on the presence and function of exosomes in edible plants. It is reported that plant cells actively secrete EVs termed PEVs under external environmental stresses like pathogenic infection. PEVs share similar morphology, release mechanisms, and content composition with mammalian/bacterial‐derived EVs. Alternatively, PEVs also contain diverse secondary metabolites not present in mammalian EVs. All these constituents jointly bestow PEVs with specific natural bioactivity.^[^ ^120^ ^]^ Moreover, PEVs have higher economic benefits and are more suitable for large‐scale production in antitumor therapy as well as in nanocarrier development due to the plant origin and abundant resources. The possible biological genesis of PEVs includes exocyst‐positive organelles, multivesicular bodies, vacuoles, and autophagosome pathways. In addition, PEV‐like biomembrane structures could also be obtained by extracting nanovesicles from the crushed liquid of plant tissues. The composition of plant‐derived biomembrane nanostructures may not be completely identical to that of their plant source. Next, we classified plants into three categories: herbs, vegetables, and fruits, and explored the applications of each type of plant in antitumor therapy (summarized in **Table** **4**).

**Table 4:** Examples of plant‐derived nanostructures employed in cancer therapy.

Types	Characteristics	Source PEVs	Engineering strategies	Nanostructures	Tumor models	Refs.
Herb	Inducing ROS	*Dendropanax morbifera*	–	Leaf‐PEVs, stem‐PEVs	–	[165]
	Macrophage polarisation					
	PDT	*Ginseng*	–	Ginseng‐PEVs	B16‐F10 melanoma	[166]
	Inducing apoptosis	*Neem, mint*, curry leaves	Chemical conjugation	EV‐CS‐GP	–	[167]
	Immunoregulation					
		*Aloe*	Coincubation	ICG/gADNVs	B16‐F10 melanoma	[168]
		*Hypericum perforatum*	–	HP‐DENs	WM266‐4 melanoma	[124]
		*Ginseng*	Membrane fusion, coextrusion	G‐EVLP‐TM	B16‐F10 melanoma, 4T1 breast tumor, CT26 colorectal tumor, MB49 bladder tumor	[127]
Vegetable	Immune stimulation	*Ginger*	–	Ginger‐PEVs	Colorectal tumor	[169]
	Macrophage polarisation	*Celery*	Co‐incubation	CELNs‐DOX	A549 lung tumor	[170]
	PDT, ICD	*Broccoli*	Transfection	EVs‐miRNA	–	[171]
	Driving innate immunity	*Cucumber*	–	CDNVs	A549 lung tumor	[172]
		*Tomato*	Sonication	Calcitriol‐TsEVs	–	[173]
		*Garlic*	–	Garlic‐EVs	B16‐F10 melanoma	[131]
		*Spinach*	Membrane fusion, coextrusion	Tk‐OMVs	CT26 colorectal tumor	[132]
		*Momordica charantia*	–	MC‐like vesicles	U251 glioblastoma	[174]
Fruit	Inducing apoptosis	*Grape*	Co‐incubation	Fisetin‐GEVs	–	[175]
	Overcoming tumor	*Grapefruit*	Conjugation	EVs‐DOX‐HR	LN229 glioblastoma	[135]
	resistance	*Pomegranate, apple, orange*	Co‐incubation	miRNA‐EVs	–	[176]
		*Bitter melon*	Sonication	5‐Fu‐EVs	CAL27 oral squamous cell carcinoma	[177]
		*Watermelon*	Co‐incubation	EVsPAMAM/miR146a	ID8/A2780/OVCAR8 ovarian tumor	[178]

### 5.1 Engineering Herbs‐Derived Nanodrugs for Cancer Therapy

Herbs, such as ginseng, lonicera japonica, and pueraria lobata, have been utilized as medicinal plant‐based remedies to cure and prevent human diseases over the past thousands of years. The therapeutic substances in herbs exert their curative effects through complex mechanisms and play an important role in safeguarding the body health. However, most of the known effective substances generally have problems such as low bioavailability, and the functions and effects of herbs are still unable to be fully revealed. With the role of PEVs in cross‐kingdom intercellular communication involving plants, animals, and microbes being discovered, studies on PEVs as a novel class of functional components existing in herbs have been increasing.^[^ ^121^ ^]^ Notably, PEVs contain the medicinally effective substances of their homologous plants, which could greatly improve the bioavailability of poorly soluble active ingredients. Furthermore, PEVs can be further modified to achieve targeting or to exert the synergistic effect of multiple components.

**Figure 7:**
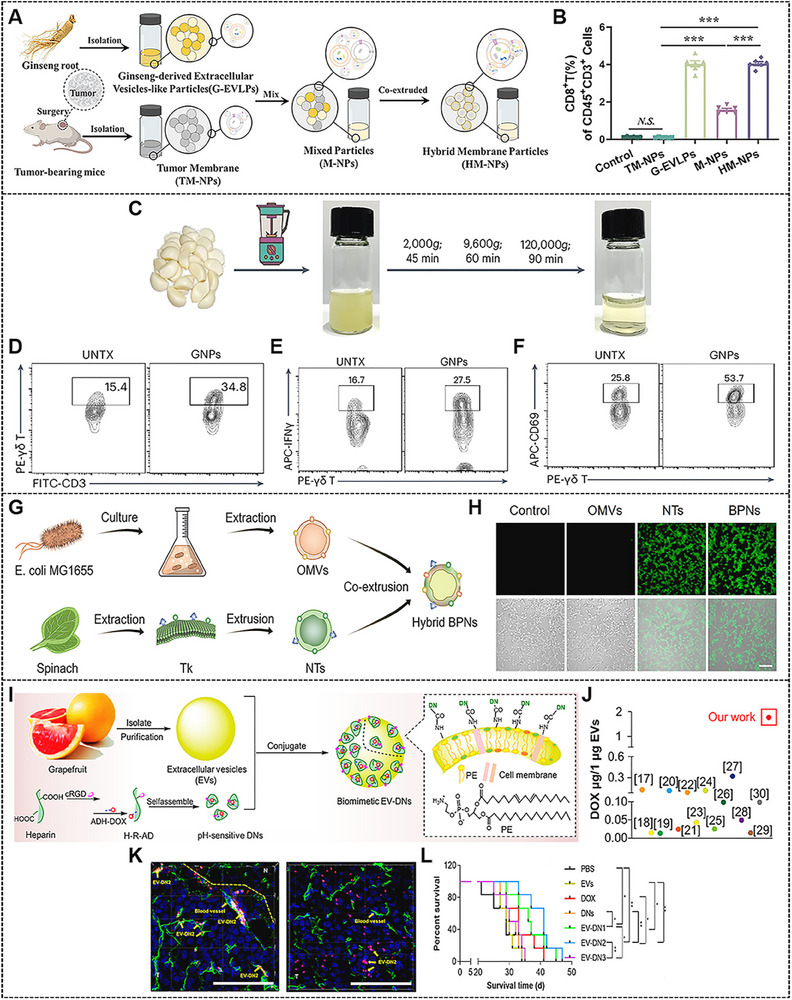
(A) Schematic diagram of the manufacturing process of functional hybrid nanovaccine. (B) The proportion of splenic CD8^+^ T lymphocytes activation after different treatment. Reproduced with permission.^[^ ^127^ ^]^ Copyright 2024, Wiley‐VCH GmbH. (C) Schematic illustration of the preparation process of garlic‐derived nanoparticles (GNPs). (D) Flow cytometric analysis of γδ T cells among CD3 cells, (E) Flow cytometric analysis of IFNγ expression in γδ T cells, (F) Flow cytometric analysis of CD69 expression in γδ T cells for the untreated group (UNTX) and GNPs groups. Reproduced with permission.^[^ ^131^ ^]^ Copyright 2024, Springer Nature. (G) Schematic illustration of preparation process of phytochemical hybrid nanovaccine. (H) Intracellular ROS production under 660 nm irradiation after different treatment. Reproduced with permission.^[^ ^132^ ^]^ Copyright 2022, American Chemical Society. (I) Schematic of the preparation for the biomimetic drug delivery system (EV‐DNs). (J) Comparison of DOX loading capacity in EVs with that in previous studies using traditional methods. (K) The 3D confocal microscopy images of glioma‐bearing brain tissues (left image, the edge of glioma; right image, the core of glioma) following intravenous injection of EV‐DNs. (L) The survival curves of mice in all groups were constructed by the Kaplan–Meier method. Reproduced with permission.^[^ ^135^ ^]^ Copyright 2021, American Chemical Society.

Accumulating evidence has indicated that numerous herbs‐derived PEVs have exhibited significant antitumor capabilities, including inducing apoptosis, triggering mitochondrial damage, suppressing cell proliferation, and hindering epithelial‐mesenchymal transition. For example, *asparagus cochinchinensis*‐derived PEVs could exert an antitumor effect in nude mice with HepG2 tumor xenografts by inhibiting the proliferation of liver cancer cells, inducing apoptosis, and upregulating the pro‐apoptotic factors. Compared with the traditional aspartate extract, the PEVs presented more distinct antitumor activity and more excellent drug‐like properties.^[^ ^122^ ^]^ Chen et al. confirmed that *tea flowers*‐derived PEVs contain large quantities of flavonoids, polyphenols, lipids, and functional proteins, which could promote the excessive generation of reactive oxygen species (ROS) to trigger mitochondrial damage and arrest cell cycle, thereby exert suppressive activities against the proliferation, migration, and invasion of breast cancer cells.^[^ ^123^ ^]^ Notably, PEVs may possess photosynthetic constituents, including chlorophyll and oxygen‐evolving complexes that facilitate ROS generation even under hypoxic conditions. For instance, PEVs derived from *Hypericum perforatum* encapsulate its principal bioactive compound hypericin, serving as a novel photosensitizer for PDT, which offers a dual‐functional platform for tumor‐targeted treatment and real‐time visualization, demonstrating substantial potential in optimizing precision oncology strategies.^[^ ^124^ ^]^ Several prominent studies emphasized the therapeutic effect of PEVs on cold tumors through immunomodulation. For example, the PEVs‐like biomembrane nanoparticles derived from *Artemisia annua* could carry plant‐derived mitochondrial DNA that was internalized into tumor‐associated macrophages (TAMs), activating the cGAS‐STING pathway and driving the transformation of pro‐tumor M2 macrophages into the antitumor M1 phenotype, thereby suppressing tumor growth and elevating antitumor immunity in a mouse model of lung cancer.^[^ ^125^ ^]^ *Brucea javanica*‐derived PEVs could deliver 10 functional miRNAs to 4T1 cells, significantly delaying the growth and metastasis of 4T1 cells by modulating the PI3K/Akt/mTOR signalling pathway and facilitating ROS/Caspase‐mediated apoptosis. Moreover, Brucea javanica‐derived PEVs were shown to inhibit the secretion of vascular endothelial growth factor and regulate the biological functions of vascular endothelial cells, which suppressed angiogenesis in the TME, ultimately inhibiting tumor metastasis and angiogenesis in 4T1 tumor‐bearing mice.^[^ ^126^ ^]^ Wang et al. elaborately developed a functional hybrid nanovaccine by integrating *ginseng*‐derived PEVs‐like nanoparticles (G‐EVLPs) with autologous tumor membranes obtained from surgically removed specimens (**Figure** **7**A). This biomimetic design significantly activated specific cytotoxic T lymphocytes through dual mechanisms (Figure 7B): G‐EVLPs‐mediated enhancement of DCs' phagocytic capacity toward autologous tumor antigens, coupled with promoting DCs maturation via the stimulation of Toll‐like receptor 4 pathway activation. This hybrid nanovaccine effectively inhibited tumor recurrence and metastatic progression across multiple models, including subcutaneous and orthotopic tumor models while establishing durable immunological memory and extending overall survival in preclinical models.^[^ ^127^ ^]^

### 5.2 Engineering Vegetables‐Derived Nanodrugs for Cancer Therapy

Fresh vegetables, such as *broccoli*, *cucumber*, and *bitter melon*, are primarily utilized in cooking or salads. They are rich in minerals, vitamins, proteins, fiber, and various other nutrients. Their constituent biomolecules, including antioxidants, polyphenols, and phytochemicals, have garnered significant scientific attention for their potential to enhance human health and mitigate the risk of chronic disease. In the daily diets, vegetables are more easily accessible and have no obvious toxicity. Reports indicated that *ginger* root comprises ≈2 × 10¹⁴ vesicles kg^−1^, which was ten times higher than the yield of mammalian‐derived EVs.^[^ ^128^ ^]^ Yang et al. isolated PEVs from *platycodon grandiflorum*, namely PGEVs, using ultracentrifugation coupled with sucrose gradient centrifugation techniques, and PGEVs promoted the polarization of TAMs toward M1 phenotype and elevated the secretion of pro‐inflammatory cytokines. Subsequent in vivo studies showed that PGEVs could efficiently accumulate in 4T1 tumors and exert substantial therapeutic effects by enhancing systemic antitumor immune responses and regulating the gut microbiota, regardless of whether they were administered orally or intravenously.^[^ ^129^ ^]^ PEVs derived from *celery* have been demonstrated to exhibit superior efficacy in tumor suppression compared to traditional synthetic carriers such as liposomes when utilized as drug delivery vehicles for DOX. Furthermore, Lu et al. revealed that *celery*‐PEVs function as immunomodulators by significantly suppressing PD‐L1 expression, thereby interfering with the PD‐L1/PD‐1 interaction and preventing T cell immunosuppression, notably, *celery*‐PEVs loaded with paclitaxel achieved a chemo‐immunotherapy combination strategy, offering a novel therapeutic approach with enhanced efficacy and reduced toxicity for lung cancer.^[^ ^130^ ^]^ A recent study discovered that *garlic*‐derived PEVs exhibit superior efficacy in activating innate immune cells (Figure 7C), particularly gamma‐delta (γδ) T cells, compared to PEVs from other edible plant sources. Oral administration of *garlic*‐derived PEVs significantly enhanced the activation of intestinal γδ T cells and subsequent IFN‐γ production (Figure 7D–F), surpassing the immunostimulatory effects observed with raw garlic consumption or isolated garlic bioactive compounds. These activated γδ T cells and IFN‐γ translocated from the gut to subcutaneous tumors, remodeling the tumor immune microenvironment and synergizing with anti‐PD‐L1 therapy to induce robust antitumor immunity.^[^ ^131^ ^]^ Especially, as a distinct type of membrane sourced from plants, TKs are equipped with diverse protein complexes, containing enzymes and photosystems that contain classic photosensitizers, chlorophyll, which executes distinct yet coordinated functions in photosynthesis. Zhuang et al. isolated TKs from *spinach* and fused them with OMVs to develop in situ tumor vaccines that possessing phytochemical characteristics for photodynamic effects‐enhanced immunotherapy (Figure 7G). The existence of TKs enables these hybrid nanovesicles to generate numerous ROS (Figure 7H), resulting in productive ICD, thereby eradicating solid tumors.^[^ ^132^ ^]^ It provides a new idea for the development of a cross‐species multifunctional membrane‐based hybrid system, which is expected to achieve efficient tumor treatment.

### 5.3 Engineering Fruits‐Derived Nanodrugs for Cancer Therapy

Extensive investigations have focused on exploring the therapeutic potential of phytochemical constituents from fruit sources in inhibiting tumor growth and suppressing metastasis. Despite promising preclinical outcomes, their translational implementation faces significant pharmacological limitations, particularly regarding bioavailability and targeted delivery. Fruit‐originated PEVs, spontaneously occurring nanovesicles, integrate the unique merits of PEVs and prospective dietary benefits as functional foods. Such dual functionality makes them serve as a revolutionary means for cancer management, since they can offer both easily accessible targeted therapeutic interventions and nutritional reinforcement. Their sustainable production feasibility and intrinsic biological compatibility highlight their potential as promising candidates for precision cancer treatment. For example, *lemon*‐derived PEVs isolated from lemon juice have been shown to upregulate the GADD45a gene through the production of ROS, leading to the S‐phase arrest and apoptosis of gastric cancer cell cycle, thus exerting anti‐gastric cancer activities in vitro and in vivo.^[^ ^133^ ^]^ Xiao et al. decorated heparin (HR)‐cRGD onto the surface of *lemon*‐derived PEVs to deliver DOX for the construction of HRED. HRED efficiently overcame DOX‐resistant ovarian cancer multidrug resistance via efficient intracellular energy depletion through diverse endocytic capabilities (caveolin‐mediated endocytosis, macropinocytosis, and clathrin‐mediated endocytosis) and remarkable reduction in drug efflux triggered by downstream production reduction of adenosine triphosphate.^[^ ^134^ ^]^ Similarly, Niu et al. patched DOX‐loaded HR nanoparticles onto the surface of *grapefruit*‐derived PEVs to construct a biomimetic drug delivery system for glioma treatment (Figure 7I). The patching strategy led to an unprecedented four‐fold enhancement in drug loading capacity compared with traditional encapsulation methods for EVs (Figure 7J). Moreover, this biomimetic nanomedicine could bypass blood‐brain barrier/blood‐brain‐tumor barrier and penetrate into glioma tissues in high abundance by HR‐mediated transcytosis and membrane fusion (Figure 7K), greatly promoting anti‐glioma efficacy in vivo (Figure 7L).^[^ ^135^ ^]^ Significantly, a thorough comprehension of the composition of PEVs is crucial for completely clarifying and further evaluating their therapeutic potential, considering that the substances contained in them govern their functions. High‐throughput sequencing techniques have been used to recognize numerous small RNA species existing in PEVs, which has promoted our acquaintance with the RNA compositions and potential influences of PEVs. Recently, Wang et al. performed a comprehensive compositional analysis of four common PEVs (grapefruit, ginger, lemon, and grape) through multi‐omics approaches to assess their potential biomedical applications. Lipidomics showed that ginger‐derived PEVs contain unique lipids, such as cytotoxic gingerol and shogaol, compared with the other three types, which remarkably inhibit tumor growth. Proteomics analysis identified 1238 common proteins and discovered three potential marker proteins, namely HSP70, Actin, and EF1α, which are also widely distributed in mammalian EVs.^[^ ^136^ ^]^ These findings emphasized the necessity of multi‐omics approaches in clarifying the possible therapeutic impacts of PEVs and in promoting the progress of innovative PEVs‐based therapies.

## 6 Current Challenges and Future Perspectives

Over the past decade, biomembrane nanostructures have emerged as a burgeoning research direction in the field of nanomedicine. Multiple‐species biomembrane nanostructures derived from mammalian cells, bacteria, and plants have enabled significant advancements in tumor treatment. However, their translation from laboratory to clinic still faces substantial challenges. In light of this, we summarized these challenges and proposed our recommendations to guide future investigations of biomembrane nanostructures.

First, in terms of clinical translation, despite promising preclinical data, only a limited number of therapies have progressed to clinical trials. One of the pioneers in the field of biomimetic nanomedicine, Academician Liangfang Zhang founded Cellics Therapeutics, which focuses on treating inflammation, viral, and bacterial infections via cell‐membrane‐coated nanoparticles. Their human RBC nanosponge (CTI‐005) has received Investigational New Drug (IND) approval from the U.S. Food and Drug Administration (FDA) for Phase I/II clinical trials against methicillin‐resistant Staphylococcus aureus infections.^[^ ^137^ ^]^ The macrophage nanosponge (CTI‐111) is also progressing toward sepsis treatment with funding support from the Combating Antibiotic‐Resistant Bacteria Biopharmaceutical Accelerator.^[^ ^138^ ^]^ Additionally, Zhang's Cello Therapeutics develops antitumor biomimetic nanosystems; its human platelet membrane‐coated cancer vaccine (CE120) has received IND approval from FDA for first‐in‐human clinical trials for multiple solid tumors, aiming to induce tumor regression and establish long‐term antitumor immunity.^[^ ^139^ ^]^ Of note, a growing number of biotechnology companies are also developing EV‐based therapies to address drug delivery challenges across various diseases. Capricor Therapeutics' exosome therapy (CAP‐1002) has completed the mid‐cycle review of FDA's Biologics License Application and is now in the final stages of the approval process. However, as documented in ClinicalTrials.gov, only 9 clinical trials were relevant to antitumor therapy, including the Gustave Roussy team's DCs‐derived exosomes in Phase II non‐small cell lung cancer treatment trial (NCT01159288). Unfortunately, clinical failures are highly prevalent, as seen in Codiak BioSciences' terminated EV trials (ExoIL‐12/STING) for solid tumors, which highlight critical bottlenecks: immunosuppressive TME, suboptimal targeting efficiency, monotherapy limitations, and so on. Moreover, bacterial‐derived OMVs are currently primarily in the preclinical research stage for antitumor therapeutic applications. As for plant‐derived biomembranes, Miller et al. conjugated curcumin with PEVs and tried to investigate the ability of PEVs to deliver curcumin to colon cancer tissue (NCT01294072). Collectively, the clinical maturity of this field remains in its infancy.

Second, concerning environmental and ethical implications, the safety issues of biomembrane nanostructures encompass both inherent material properties and contaminant‐derived risks (including but not limited to mycoplasma, viruses, endotoxins, and culture medium components). Functional heterogeneity of CMs/EVs demands thorough characterization, especially given evidence that cancer cell‐derived EVs may paradoxically accelerate malignancy. Simultaneously, residual contaminants could trigger adverse responses, emphasizing the critical need for standardized quality control in biomembrane therapeutic manufacturing. While mammalian cell‐derived biomembrane nanostructures benefit from inherent biocompatibility, their clinical translation faces immunogenic and production hurdles. To address this, parent cells must be secured from a healthy source (autologous or universal cell lines) to eliminate the risk of infectious diseases. Bacterial‐derived biomembrane nanostructures show unique targeting and immunomodulatory functions, demanding stringent endotoxin management. In contrast, plant‐derived biomembrane nanostructures, despite advantages in wide sources and scalable production, require genetic engineering to mitigate cross‐species immune recognition. Finally, while current studies predominantly focus on short‐term toxicity (≤28 days), chronic toxicity gaps are critical, with ≥6‐month primate data urgently needed to assess neurotoxicity and reproductive toxicity. Substantial interspecies differences limit extrapolation of animal safety data to humans. Therefore, comprehensive safety validation remains imperative before clinical applications.

Third, regarding technical bottlenecks, as a novel therapeutic modality, biomembrane‐based nanomedicine still requires further optimization in technical details for practical applications. The functionalization strategy is directly related to technical challenges. When preparing biomembrane‐coated nanoparticles through physical modification methods (ultrasonication and coextrusion), more than 90% of resulting membrane‐coated nanoparticles are only partially coated, with only ≈40% of these partially coated nanoparticles being internalized by source cells, resulting in suboptimal targeting ability.^[^ ^140^ ^]^ The underlying mechanisms governing how original cell membranes rupture and subsequently fuse to result in partial coating remain poorly understood. Notably, Liu et al. discovered that the membrane fluidity is a key factor regulating the fixation of partial coating and employed exogenous phospholipid as a fluidity‐enhancing helper to promote the fusion of lipid patches, achieving a ≈4‐fold improvement in full coating ratio. Consequently, a thorough understanding of the dynamic membrane rupture‐fusion process, including its specific steps and key regulatory parameters, is essential for developing more efficient full‐coating technologies and represents a pivotal direction for future precision biomimetics.^[^ ^141^ ^]^ Chemical modification methods usually require specific reaction conditions, such as pH, temperature, chemical reagents, and so on, which may disrupt biomembrane activity, including the destruction of membrane structure, abnormal aggregation of membrane proteins, and unexpected exposure of phosphatidylserine, resulting in unpredictable damage of membrane integrity. Consequently, the modification strategy should be carefully selected according to specific application scenarios, and specific chemical modification methods that precisely regulate the reaction amount and localization will minimize detrimental effects on biomembrane activity and function. Moreover, chemical modification methods increase the immunogenicity of engineered biomembranes in the circulation, which might trigger immune cell‐mediated clearance and reduce the delivery efficiency.^[^ ^142^ ^]^ It is imperative to develop immunocompatible molecular modifications through adaptive molecular engineering. Biological modification methods, especially genetic engineering, offer inherent advantages as dual‐purpose carriers for both protein and nucleic acid therapeutics. However, genetically engineered biomembranes expressing specific proteins are prone to rapid hepatic clearance in vivo, significantly limiting their therapeutic targeting efficacy.^[^ ^143^ ^]^ Consequently, selecting low immunogenic cells as donor cells (e.g., MSCs) or using genetic engineering to knock out immunogenic factors might unlock the long‐term application potential of biomembrane in cancer treatment.

Fourth, regarding emerging trends, systematic investigation is needed to elucidate the physiological functions of biomembranes across species, along with establishing a dedicated database for improved data accessibility. Comprehensive characterization of biomembrane molecular composition through multi‐omics analyses is essential to understand their interactions with TME, antitumor mechanisms, safety profiles, and to establish production guidelines. Then, rational assembly of cross‐species biomembrane components can then generate functionally enhanced nanoarchitectures with synergistic antitumor effects. Specifically, tailored biomembrane nanomedicines could be developed by integrating patient‐specific tumor genomic profiles and TME characteristics, such as screening optimal membrane surface modification targets through single‐cell sequencing.

## 7 Summary

The precise biodistribution of nanomedicine delivery systems depends fundamentally on active biomolecular recognition mechanisms. While traditional synthetic nanocarriers could be engineered with tunable physicochemical parameters or programmed with stimulus‐responsive functionalities to optimize therapeutic payload delivery. Existing approaches in active targeting nanomedicine remain constrained by the current inability to fully address the multifaceted biological interactions. Mammalian cells/bacteria/plants‐derived biomembrane nanostructures exhibit a high degree of heterogeneity and complexity in structure and function, which present vital inherent class properties for developing precision nanomedicines. For example, transferring the complexity and dynamism of biomembranes to synthetic nanocarriers could provide the ability to overcome biological barriers and maintain a great tendency for tumor targeting delivery. Moreover, such biomimetic interfaces could also harness their inherent biological functions and properties to more feasibly reshape the TME, achieving antitumor therapy. Taken together, the biomembrane engineering paradigm, leveraging evolutionarily conserved membrane architectures, holds significant promise for developing next‐generation therapeutic vectors with enhanced biofunctionality for precision oncology and translational medical innovations.

## Conflict of Interest

The authors declare no conflict of interest.

## Author Contributions

X.W. was responsible for conceptualization, methodology, and visualization; wrote the original draft, edited the final manuscript and secured funding. C.W., X.C., Y.Z., M.Y., N.L., and H.H. helped collect the information. G.Z. secured funding. M.Z. reviewed and edited the final manuscript. Y.L. supervised the work, secured funding, acquired resources, and reviewed the final manuscript. R.G. led the supervision, oversaw funding and resource acquisition.
